# Using risk of bias domains to identify opportunities for improvement in food- and nutrition-related research: An evaluation of research type and design, year of publication, and source of funding

**DOI:** 10.1371/journal.pone.0197425

**Published:** 2018-07-05

**Authors:** E. F. Myers, J. S. Parrott, P. Splett, M. Chung, D. Handu

**Affiliations:** 1 EF Myers Consulting Inc, Trenton, Illinois, United States of America; 2 Departments of Interdisciplinary Studies and Nutritional Sciences, Rutgers University, Newark, New Jersey, United States of America; 3 Splett & Associates, LLC, Stanchfield, Minnesota, United States of America; 4 Department of Public Health and Community Medicine, Tufts University School of Medicine, Boston, Massachusetts, United States of America; 5 Research International and Scientific Affairs, Academy of Nutrition and Dietetics, Chicago, Illinois, United States of America; University of Ioannina Medical School, GREECE

## Abstract

**Purpose:**

This retrospective cross-sectional study aimed to identify opportunities for improvement in food and nutrition research by examining risk of bias (ROB) domains.

**Methods:**

Ratings were extracted from critical appraisal records for 5675 studies used in systematic reviews conducted by three organizations. Variables were as follows: ROB domains defined by the Cochrane Collaboration (Selection, Performance, Detection, Attrition, and Reporting), publication year, research type (intervention or observation) and specific design, funder, and overall quality rating (positive, neutral, or negative). Appraisal instrument questions were mapped to ROB domains. The kappa statistic was used to determine consistency when multiple ROB ratings were available. Binary logistic regression and multinomial logistic regression were used to predict overall quality and ROB domains.

**Findings:**

Studies represented a wide variety of research topics (clinical nutrition, food safety, dietary patterns, and dietary supplements) among 15 different research designs with a balance of intervention (49%) and observation (51%) types, published between 1930 and 2015 (64% between 2000–2009). Duplicate ratings (10%) were consistent (κ = 0.86–0.94). Selection and Performance domain criteria were least likely to be met (57.9% to 60.1%). Selection, Detection, and Performance ROB ratings predicted neutral or negative quality compared to positive quality (p<0.001). Funder, year, and research design were significant predictors of ROB. Some sources of funding predicted increased ROB (p<0.001) for Selection (interventional: industry only and none/not reported; observational: other only and none/not reported) and Reporting (observational: university only and other only). Reduced ROB was predicted by combined and other-only funding for intervention research (p<0.005). Performance ROB domain ratings started significantly improving in 2000; others improved after 1990 (p<0.001). Research designs with higher ROB were nonrandomized intervention and time series designs compared to RCT and prospective cohort designs respectively (p<0.001).

**Conclusions:**

Opportunities for improvement in food and nutrition research are in the Selection, Performance, and Detection ROB domains.

## Introduction

How much trust or confidence can be placed in food and nutrition research results? This is an important question for the public, those conducting systematic reviews, as well as those using research to inform policy and practice. Much of this dialogue about trusting research findings has focused on the potential for industry funding to bias research results to be more favorable to their interests [1]. A recent series of articles in *JAMA* on conflict of interest highlighted this concern with the following statement: “The challenges involving the role of the food industry in research are profound and not easily dismissed” [2–4]. More recent dialogue suggests that the use of the source of funding as a proxy for risk of bias (ROB) is insufficient and there is a call for a broader definition of conflict of interest, which underscores research integrity as the foundation for valid study findings [5].

A 2015 Institute of Medicine report, *Trust and Confidence at the Interfaces of the Life Sciences and Society*: *Does the Public Trust Science*?, acknowledged that there are many factors that affect the public trust, including the need for members of the scientific community itself to shift their values away from “outcomes of research” and focus more on the process of research [6]. Focusing on the process of research includes the concept of responsible conduct of research and reduction of the ROB in research. ROB is defined as the possibility of systematic error or deviations from the truth in inferences of findings [7, 8]. In a recent report titled *Fostering Integrity in Research*, the National Academies of Sciences, Engineering, and Medicine concluded that the following core values help ensure that the research enterprise advances knowledge: objectivity, honesty, openness, fairness, accountability, and stewardship [9]. The report concluded that “Integrity in science means planning, proposing, performing, reporting, and reviewing research in accordance with these values” [9].

Individuals who conduct systematic reviews are acutely aware of the need to assess the ROB to inform the amount of confidence that can be placed in research findings [10]. Systematic reviews have become common in many fields, including the food and nutrition community, and are considered the optimal way to synthesize the body of research to address a specific issue [4]. Key elements of a systematic review are the critical appraisal of each research study and assessment of the ROB to inform the amount of confidence that can be placed in the body of research findings included in the systematic review [11, 12].

Methods of critically appraising research included in systematic reviews continue to evolve. The Agency for Healthcare Research and Quality (AHRQ) summarized the factors that should be included in 2002 [12]. Tools continue to be developed to assist in the appropriate synthesis of a body of research into a characterization of the “strength” of the evidence [11, 13, 14]. The Cochrane Collaboration’s methodology for systematic reviews foreshadowed the most recent dialogue by shifting their evaluation of risk of bias to focus on the following five ROB domains: Selection, Performance, Detection, Attrition, and Reporting [8, 11]. Dialogue about whether the source of funding should be explicitly included in the ROB domains is ongoing; however, to date, the focus has remained on the actual merits of the research design, conduct, and reporting, rather than the source of funding itself [15–18]. In addition, there may be a return to the inclusion of an overall rating that summarizes the individual ROB domain ratings [19]. With the exception of one recent research study, the concept of evaluating ROB as a way to identify improvements needed in a body of research has not yet been reported [20].

Leading government and professional organizations such as the US Department of Agriculture (USDA), the AHRQ, and the Academy of Nutrition and Dietetics conduct and use systematic reviews based on the best nutrition research available to inform public policy, dietetics practice decisions, and research agenda development [14, 21, 22]. Those organizations, as well as the entire food and nutrition research community, rely on the availability of research that meets high standards of scientific integrity and has low risk of bias.

There has not yet been a comprehensive assessment within the body of food- and nutrition-related research to determine the degree to which ROB domain criteria are being met. Such knowledge would expand the dialogue about ROB and help identify opportunities for strengthening future food and nutrition research.

This retrospective cross-sectional study was designed to investigate ROB identified in studies utilized in systematic reviews for food- and nutrition-related topics conducted by three major organizations that produce systematic reviews (USDA, AHRQ, and the Academy of Nutrition and Dietetics). Specifically, the following three questions were addressed:

1. Are critical appraisal ratings similar for the same article if more than one critical appraisal rating has been completed in the different systems?
2. Which of the ROB domain ratings best predicted the overall quality rating assigned to the research article?
3. What opportunities for improvement in food and nutrition-related research can be identified by the degree that funder, research type and design, and publication year predict the ROB domain ratings?

## Materials and methods

### Sample

The sample for this study consists of critical appraisal records of food- and nutrition-related research articles included in systematic reviews available on the websites of the following three organizations in August 2016:

- Academy of Nutrition and Dietetics Evidence Analysis Library (EAL) (all systematic reviews conducted from 2004 to 2016; same tool used for all appraisals) [13]
- AHRQ Evidence-Based Practice Center Reports (nutrition-related projects conducted from 2004 to 2016; appraisal tools specific to each project) [21]
- USDA Nutrition Evidence Library (NEL) (all systematic reviews conducted from 2010 to 2015; two tools used for appraisals) [23]

**Fig 1:**
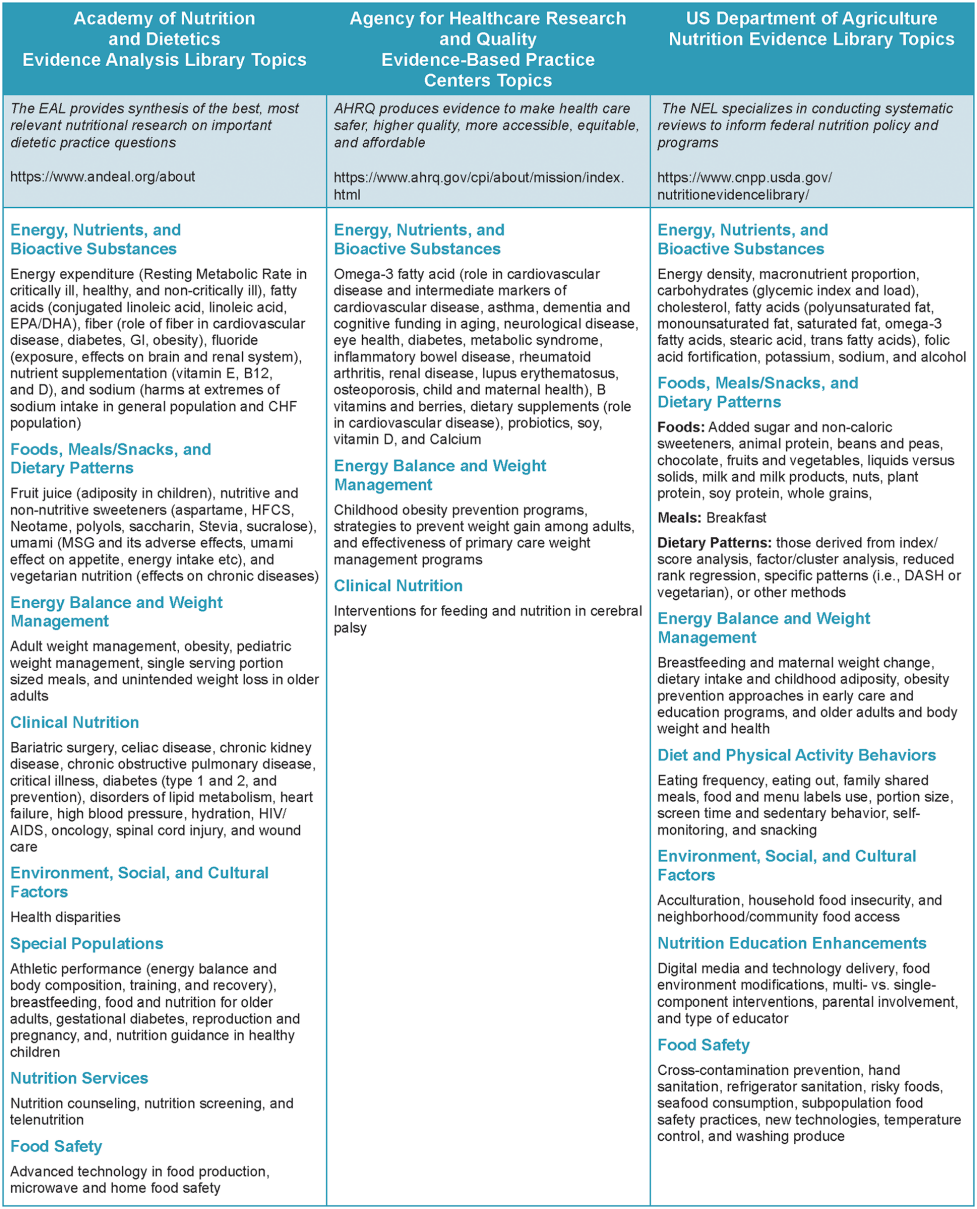
Nutrition topics included in systematic reviews conducted by three organizations.

These organizations (or data sources) have different missions that are reflected by the purposes of the systematic reviews, variation in the topics, and characteristics of the research reviewed as shown in Fig 1. Thus, the resulting database includes a wide range of applied, human food and nutrition research. The majority of topics addressed by the EAL were clinical or treatment oriented; the topics included in the AHRQ reports focused predominantly on nutrients or dietary supplements and relationship with disease markers, while topics addressed by the NEL focused primarily on food-based intake and chronic disease prevention. Search criteria and critical appraisal tools used by each organization reflect their purpose, topics, and types of research reviewed and have evolved over time [13, 14, 24]. (See S2 and S3 Figs) The appraisal tools can be found in the EAL and NEL database and within each AHRQ evidence report [13, 14, 21]. In the EAL and NEL, common critical appraisal methods were used for all projects. However, for AHRQ systematic reviews, critical appraisal methods and tools were selected or developed by the research team for each report or topic. All appraisals had been previously completed by trained personnel following established procedures and supervised by project managers within each of the three organizations. Each organization provided access to critical appraisal records of published research reports used for secondary analysis in this research. There were no human or animal subjects and IRB approval was not sought.

The cut-off date for including reviews in the sample was established as the beginning of the data extraction process in August 2016 when appraisal files were transmitted to the researchers. Critical appraisal records were screened for completeness of data and duplication across or within data sources (see S1 Fig).

### Variables

**Fig 2:**
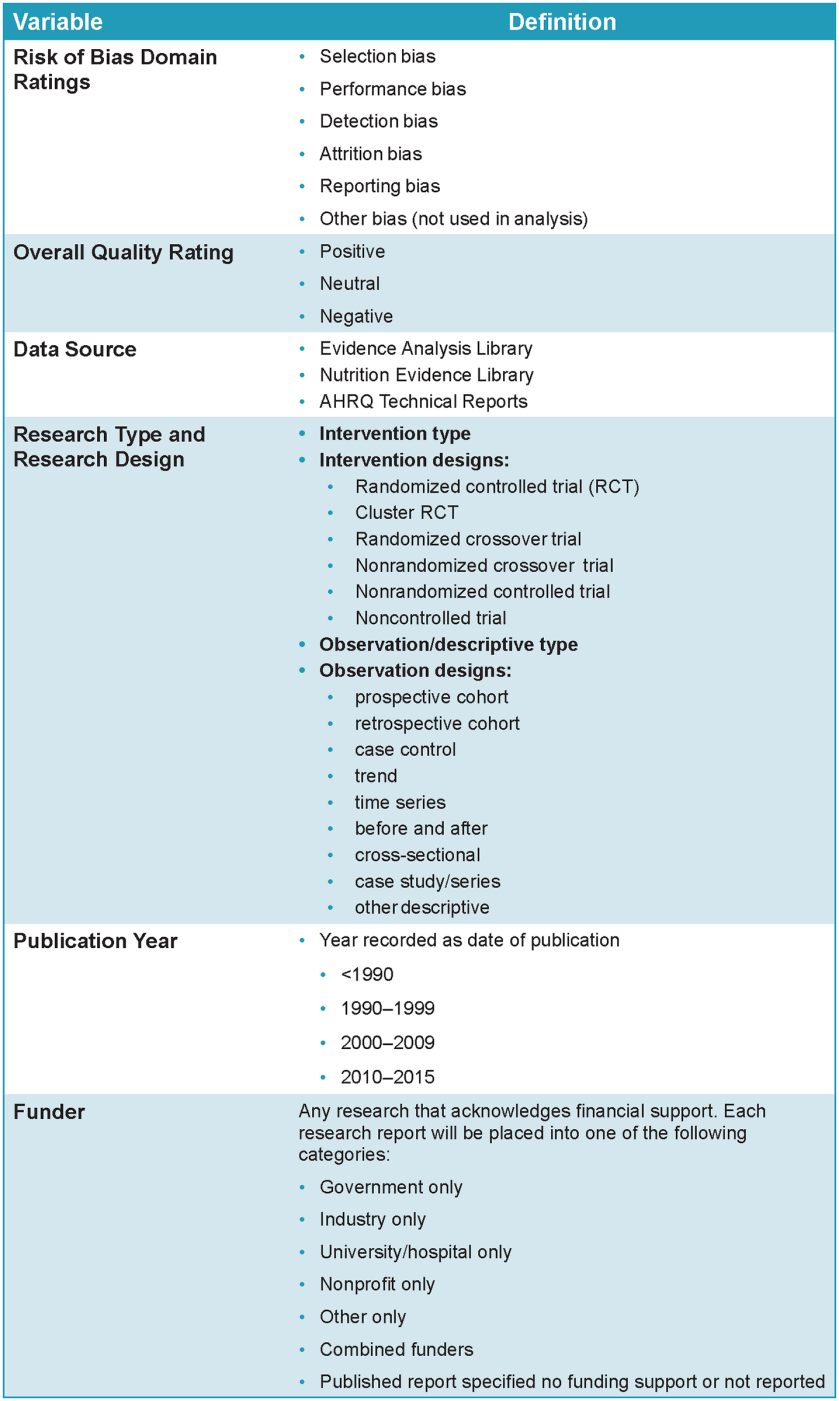
Study variables. *Publication years were selected based on decades prior to reporting guidelines (<1900 and 1900–1999), decade of implementing publication guidelines (2000–2009), and after implementation of publication guidelines (2010–2015). AHRQ, Agency for Healthcare Research and Quality; RCT, randomized controlled trial.

Fig 2 describes the study variables: data source (EAL, NEL, or AHRQ), ROB domains, overall quality rating (negative, neutral, or positive), type of research (intervention or observational) and specific research design, publication year, and funder.

The five ROB domains defined by the Cochrane Collaboration (Selection, Performance, Detection, Attrition, and Reporting) were selected because of their wide use and acceptance [10, 11]. The global overall quality rating was available only in the EAL and some AHRQ reports; and a subset of early AHRQ projects only used an overall quality rating and did not provide specific ROB criteria ratings. Funder categories were identical to those used in an earlier study [1]. Funder was not included in the critical appraisal records of most AHRQ records; if missing, these funder data were manually retrieved from published research articles by research staff and coded.

### Mapping of appraisal questions to ROB domains

**Fig 3:**
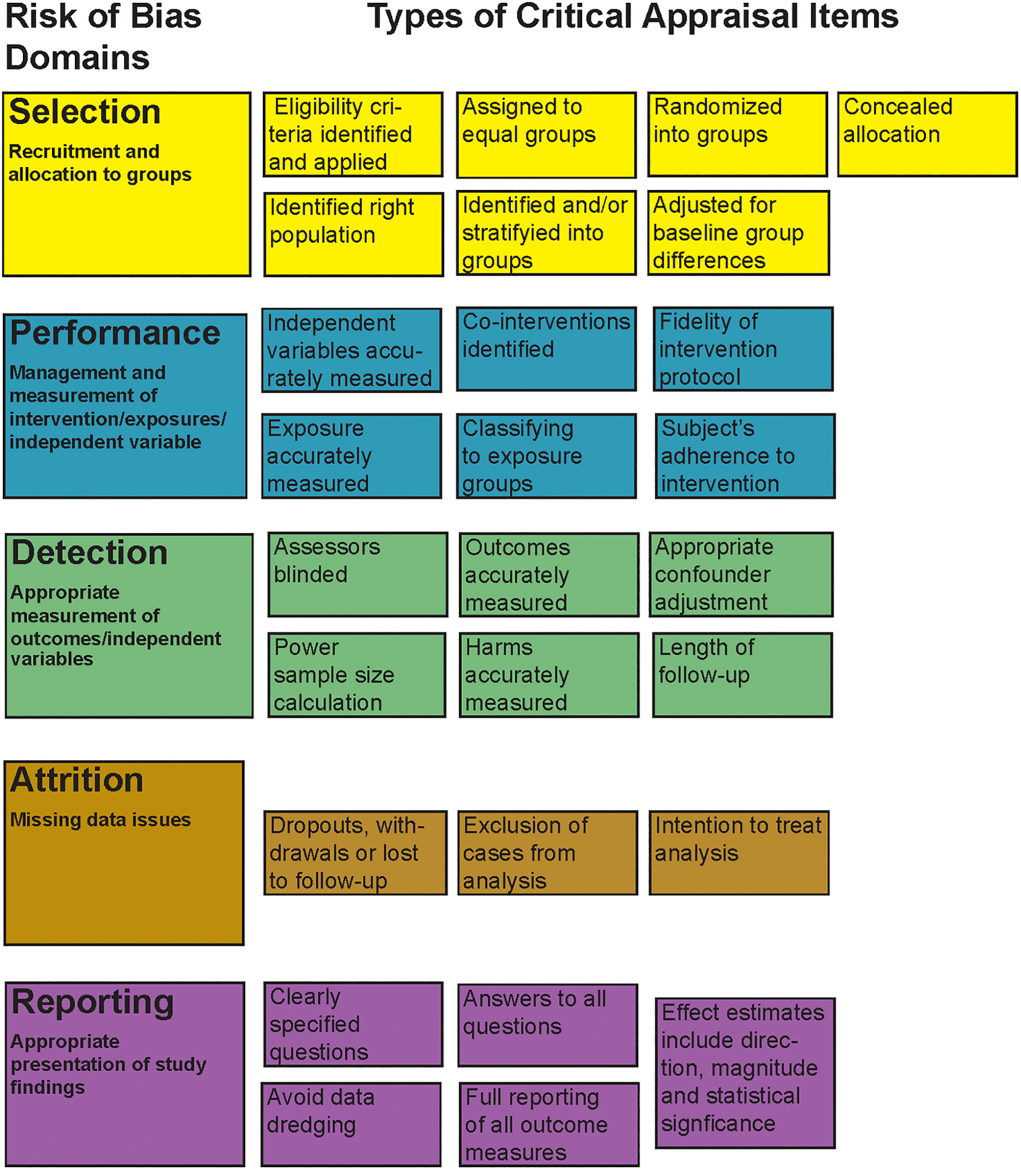
Mapping critical appraisal items to risk of bias domains.

Using original quality appraisal tools employed in each project, the research team mapped quality criteria questions used in the appraisal tools to the five ROB domains. To develop the methodology for this, each member of the research team independently submitted an initial alignment of individual questions to ROB domains, differing alignments were discussed, and final mapping of questions was reached by consensus. Through this process, appraisal criteria questions for both interventional and observational studies were mapped to the five ROB domains. Fig 3 describes each domain and shows the types of critical appraisal items mapped to it for this study. While the Cochrane ROB domains were primarily developed for randomized controlled trials (RCTs), criteria proposed for observational studies closely match those domains [8, 25].

### ROB ratings

ROB domain ratings were calculated for each data source in the following manner. Analysts had previously assigned quality criteria questions the following ratings: yes (criterion met), no (criterion not met), unclear, undetermined, or not applicable.

For coding purposes, ratings of “no”, “unclear”, and “not determined” were collapsed into “no” (indicating that the validity criterion was not obviously met). Thus, “yes” responses were coded 1 and “no/uncertain” responses were coded 0. “Not applicable” responses were treated as missing (i.e., did not count toward a study’s ROB rating).

Because multiple criteria (questions) could be mapped to a ROB domain, rating responses within each domain were averaged (values 0 to .5 were coded as 0, and values greater than .5 were coded as 1). This resulted in domain scores between 0 and 1 (where 0 indicates that the criterion for the domain was not met and 1 indicates that it was).

In the AHRQ data set, a single article could have multiple ratings, due to the practice of rating the article for the specific systematic review topic. Critical appraisal methods and tools were selected or developed by the research team for each report or topic. Discrepant ROB ratings for the same article were, in fact, rare (ranging from .6% of articles for Performance to 3% of articles for Detection). To prevent the multiple entries in the AHRQ data set from greater weight relative to the other data sets, multiple ratings were resolved using the following principles: (1) the most common rating was accepted (e.g., for three instances with the same rating and one different, the value of the three was accepted), and (2) in the 1.1% of cases (n = 18) in which there was a tie, the higher or lower rating was selected alternatively (to avoid a consistent bias up or down). This same procedure was used to reconcile the discrepant ratings when articles were included in more than one data source.

### Statistical analysis

Descriptive statistics were computed for all variables. Bivariate associations between funder, study design, and publication year were calculated using chi-square tests and standardized residuals were examined to identify cells where observed frequencies departed from expected.

#### Comparison of critical appraisal ratings for the same research article (question 1)

To evaluate the consistency of ROB rating across organizations/sources, the kappa statistic, with p<0.05 was used to assess the level of agreement (consistency) in a subset of data in which ROB domain ratings for the same research article were available from more than one critical appraisal record.

#### Prediction of the overall quality ratings by ROB domain ratings (question 2)

Multinomial logistic regression, including research type and design, was used to examine the association between not specific ROB domain ratings and overall quality ratings (neutral versus positive and negative versus positive) in the subset of data with overall study quality ratings. These models were adjusted for publication year category.

#### Prediction of ROB domain ratings by publication year, funding source, and research design/type (question 3)

Using the combined dataset, binary logistic regression was used to evaluate the association between funder and ROB domain ratings within research type (intervention and observational) after adjusting for publication year and specific research design. An exploratory analysis that included data source as an additional co-variate was used to examine the impact of data source on the prediction of ROB domain. Because of multiple analyses, a Bonferroni correction was applied and the a priori alpha was set at p<0.005.

## Results

### Description of sample

**Fig 4:**
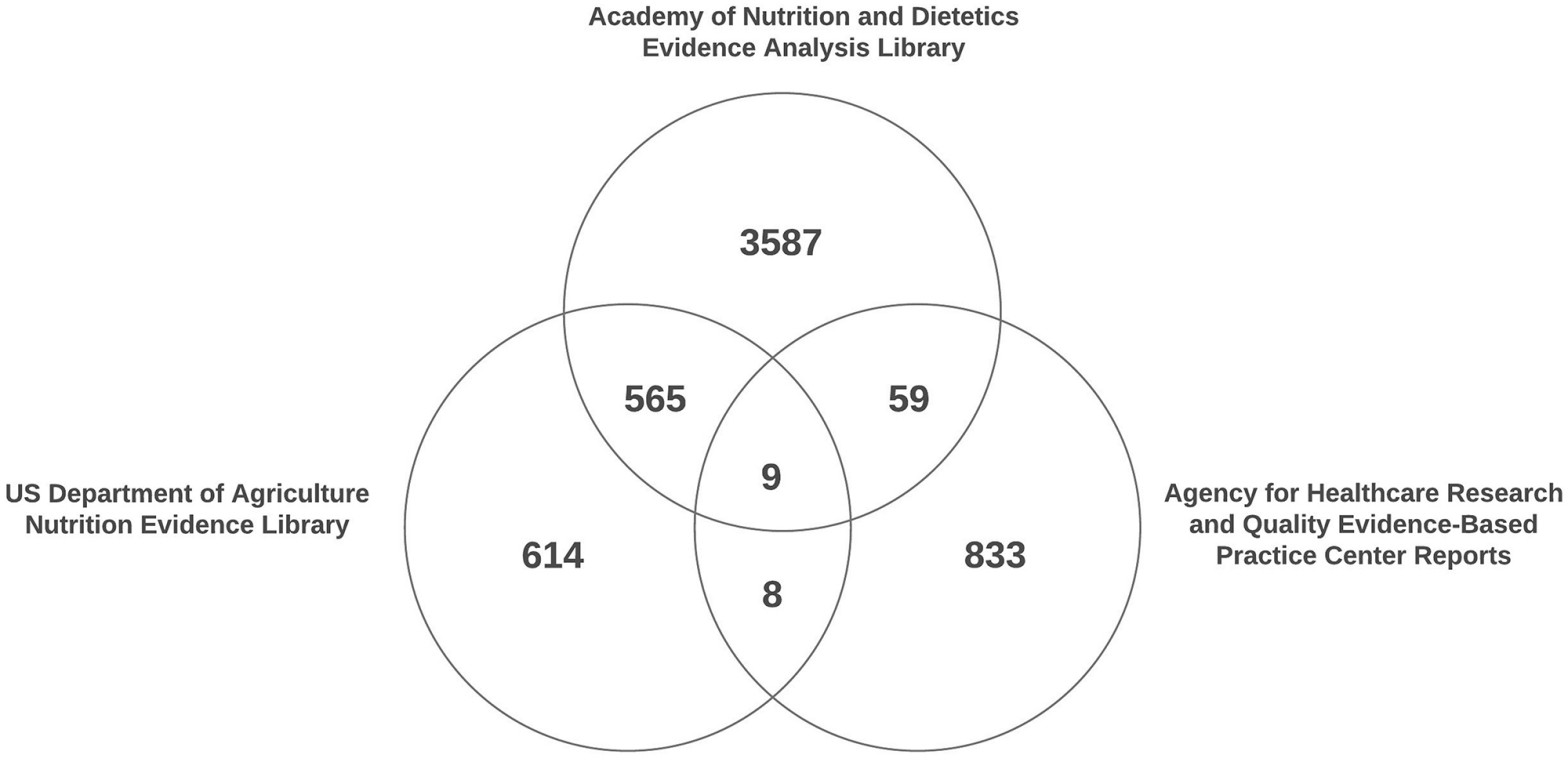
Distribution of sample of critical appraisal records. *Critical appraisal records for research articles included in systematic reviews in each system. A single article may have critical appraisal records in more than one AHRQ report or records within more than one system.

Critical appraisal records for a total of 5675 unique studies were available for analysis. Fig 4 presents the number of studies with critical appraisal records that are unique to each data source and are included in more than one data source. Very few studies (n = 9) had critical appraisal ratings in all three data sources. The largest overlap in studies was between the EAL and the NEL, with 574 articles with critical appraisal ratings in both systems. Descriptive statistics on funding source, study design, year, overall quality ratings, and ROB domain ratings are presented in Table 1.

**Table 1:** Characteristics of food and nutrition research used in systematic reviews.

Characteristic	n	%
Funder		
Government only	1355^a^	23.9
Industry only	461	8.1
University only	958	16.9
Nonprofit only	337	5.9
Other only	264	4.7
Combined funders	1455	25.6
Not reported or no funding	845	14.9
Total	5675	100.0
Study design		
Interventional designs^a^		
Randomized controlled trial (RCT)	1888	65.0^c^
Cluster RCT	45	1.5
Randomized crossover trial	21	0.7
Nonrandomized crossover trial	540	18.6
Nonrandomized controlled trial	382	13.2
Noncontrolled trial	28	1.0
Total	2904	100.0
Observational designs^a^		
Prospective cohort	1016	36.7
Retrospective cohort study	177	6.4
Case control study	290	10.5
Trend study	34	1.2
Time series	87	3.1
Before-after study	121	4.4
Cross-sectional study	939	33.9
Case study or case series	42	1.5
Other descriptive	65	2.3
Total	2771	100.0
Overall quality rating^b^		
Positive	2274	47.8
Neutral	2183	45.9
Negative	303	6.4
Total	4760	100
ROB domain criteria met^c^		
Selection	5504	57.9
Performance	5406	60.1
Detection	5462	75.2
Attrition	4744	79.7
Reporting	5007	84.7
Year		
<1990	223	3.9
1990–1999	1073	18.9
2000–2009	3645	64.2
2010–2015	734	12.9

Sample drawn from EAL, NEL and AHRQ Reports. AHRQ, Agency for Healthcare Research and Quality Evidence-Based Practice Center Reports; EAL, Academy of Nutrition and Dietetics Evidence Analysis Library; NEL, US Department of Agriculture Nutrition Evidence Library; RCT, randomized controlled trial; ROB, risk of bias.

^a^Percentages of study designs are within design type.

^b^Percentages are of the total sample with quality ratings.

^c^Percentages are of the total sample with specific ROB domain ratings. Some ROB domains are not included in specific research designs or in AHRQ reports so n varies by domain.

#### Funder

Combined funders (n = 1455, 25.6%) and government-only funders (n = 1355, 23.9%) were the most frequently indicated funding sources in the sample (Table 1). Nonprofit-only funders (n = 337, 5.9%) and other-only funders (n = 264, 4.7%) were the least frequently indicated funding sources. Nearly 15% of the included studies (n = 845) had no funder indicated or explicitly reported no funding for the study.

#### Research type

The sample was almost equally split in terms of observational (n = 2904, 51.2%) and interventional (n = 2771, 48.8%) types of research. The distribution of study designs is shown in Table 1.

The most predominant type of study designs overall were RCTs (33%), prospective cohort (17.9%), and cross-sectional (16.5%).

#### Association between funding source and research design

**Fig 5:**
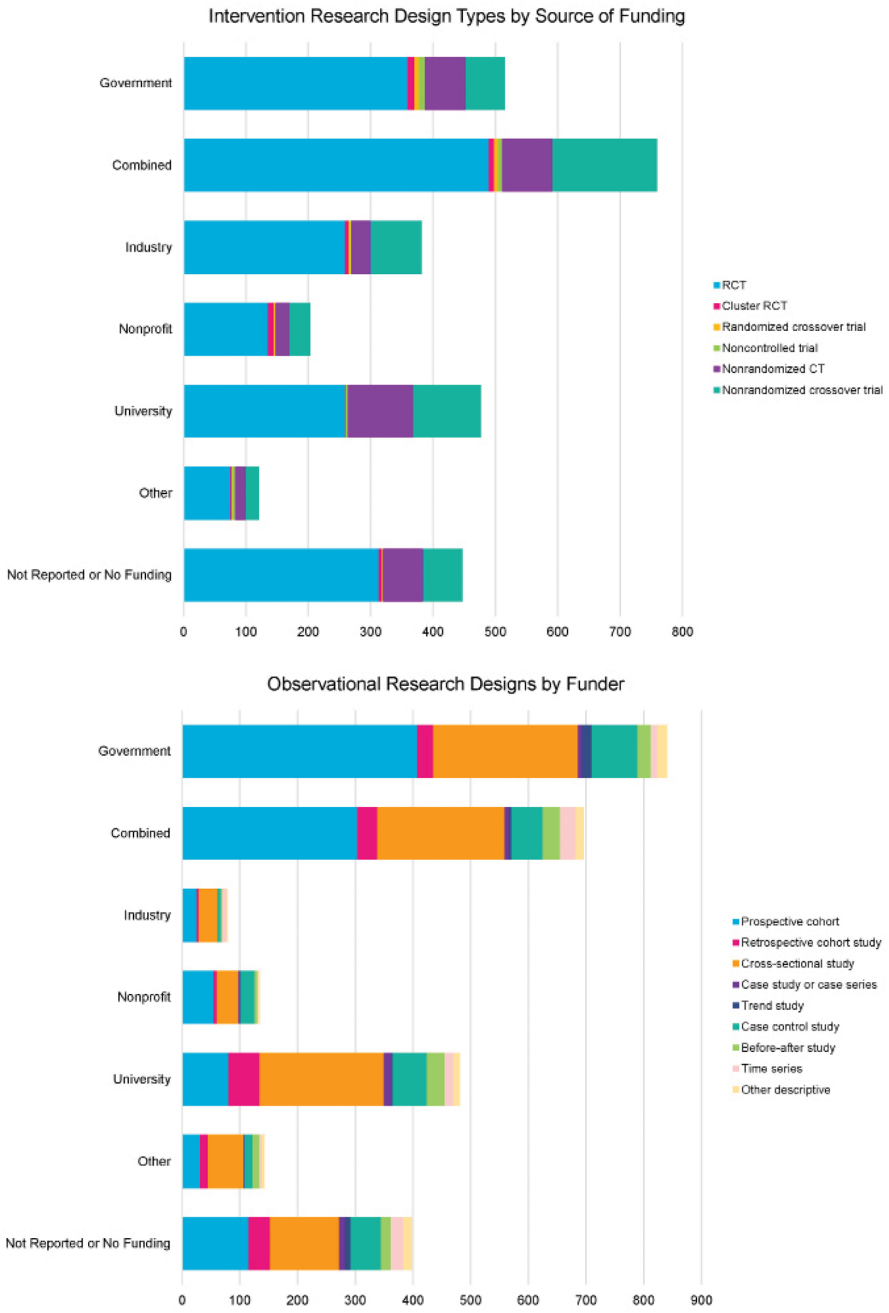
Research design types by source of funding. CT, controlled trial; RCT, randomized controlled trial.

There was a significant association between funding source and study design for both interventional and observational study design types (p<0.001 for both) (Fig 5; data in S1 Table). The proportion RCTs was similar for across all funders (62.0%–70.0%) with the exception of university-only funders (n = 257, 54.1%). University-only funders were less likely to fund RCT designs (z = −3.0, p = 0.001) or cluster RCTs (z = −2.0, p = 0.02) and were more likely than expected to fund nonrandomized designs (nonrandomized controlled trial: z = 5.2, p<0.001; nonrandomized crossover trials: z = 2.2, p = 0.01).

For observational designs, prospective cohort designs were *more* likely to be funded by government-only funders (48.5%, z = 5.6, p<0.001) and combined funders (43.7%, z = 3.1, p<0.001) and were less likely to be funded by university-only (z = −7.3, p<0.001) or other-only (z = −3.0, p = 0.001) funders or to have no funder indicated or explicitly report no funding (z = −2.6, p = 0.005).

#### Publication year

Critical appraisal reports evaluated studies from 1930 to 2015. The largest proportion (64.2%, n = 3645) of studies included across data sources were published in the decade 2000–2009 (Table 1).

#### Quality ratings

A subset of data (n = 4760) included an overall quality rating (positive, neutral, or negative) in the critical appraisal reports. Positive ratings were reported for 47.8%, neutral ratings for 45.9%, and negative ratings for 6.4% (Table 1).

#### ROB domain rating criteria met

Table 1 provides the frequency and percentage of studies meeting ROB criteria across the five domains. Domains with the lowest proportion of studies meeting ROB criteria and the highest risk of bias were Selection (57.9%, n = 3186) and Performance (60.1%, n = 3251). Proportions of studies meeting ROB criteria for each specific research design are included in the S2 Table.

### Findings from ROB analyses

#### Consistency of individual research report ROB ratings between data sources (question 1)

About 10% of articles (n = 641) had appraisal records in more than one system. This provided some potential for assessing consistency of ratings across systems. Very high agreement was found between EAL Quality Criteria Checklist and NEL Research Design and Implementation ratings for all ROB domains (κ = 0.86–0.94 for the different domains), in which 574 overlapping records were available for analysis. However, for the other systems, the number of overlapping records between other systems was very small and was not statistically significant.

#### Relationship between ROB domain ratings and overall quality rating (question 2)

In order to predict overall quality rating from the five ROB domains, each case had to have both a quality rating and all ROB domain ratings. There were 3873 cases available for this analysis. Domain effects when predicting overall quality rating were adjusted by category of year published and funder. Adjusted domain effects on overall quality are reported separately for the sample stratified by research type (Table 2). All ROB domains were significantly associated with an increase in the likelihood of receiving a negative versus a positive rating in either interventional or observational research if they were unmet. The three domains that were consistently significant in both types of research and for both neutral and negative overall quality ratings were Selection, Performance and Detection. Significant odds ratios for these three domains ranged from a high for the Selection domain in interventional research of OR = 84.68 (p<0.001) to OR = 1.96 for the Performance domain in observational research. The highest significant odds ratios were for the Selection domain for those receiving a negative or neutral overall quality rating in interventional research (OR = 150.11 and 84.60 respectively, p<0.001) and for those receiving a neutral rating in observational research (OR = 63.89 p<0.001).

**Table 2:** Summary of significant relationships between overall quality and ROB domain ratings by research type^a^.

		Interventional				Observational			
		OR	95% Confidence Interval for OR		Sig.	OR	95% Confidence Interval for OR		Sig.
			Lower Bound	Upper Bound			Lower Bound	Upper Bound	
Negative*	Selection	150.11	59.79	376.91	<0.001	39.70	20.93	75.32	<0.001
	Performance	9.04	4.03	20.25	<0.001	10.50	5.24	21.05	<0.001
	Detection	40.49	19.53	83.95	<0.001	36.00	19.38	66.86	<0.001
	Attrition	4.81	2.43	9.52	<0.001	1.90	1.00	3.64	0.052
	Reporting	1.60	0.80	3.23	0.186	10.35	5.41	19.81	<0.001
Neutral	Selection	84.68	60.02	119.47	<0.001	63.89	43.75	93.28	<0.001
	Performance	2.12	1.54	2.92	<0.001	1.96	1.43	2.70	<0.001
	Detection	10.64	7.01	16.14	<0.001	19.40	13.10	28.72	<0.001
	Attrition	1.15	0.78	1.71	0.483	1.18	0.75	1.86	0.479
	Reporting	0.71	0.46	1.08	0.11	1.54	0.95	2.49	0.08

*Models adjusted for funding source and year published.

CI, confidence interval; OR, odds ratio; ROB, risk of bias.

^a^All models were adjusted for year, funder, and study design type. Three overall quality ratings were possible: positive, neutral, and negative. The comparator used for this analysis was the likelihood of receiving either a neutral or negative rating compared to a positive rating if the ROB domain rating was *not* met, e.g. higher risk of bias for domain led to lower overall quality rating.

#### Final predictive model for meeting ROB domain ratings (question 3)

The two domains least likely to meet the ROB criteria (thus, have a highest risk of bias) were Selection and Performance (see Table 1). However, more information is needed in order to understand what factors may contribute to studies having greater risk of bias. Initial bivariate analyses indicated that funder, publication year, and specific study design were all significantly associated with whether ROB criteria were met in most domains (p<0.05 for funder except for Attrition; p<0.05 for year for all domains; p<0.05 for study design for all domains). In addition, funder and study design, as well as funder and year published and study design and year published, were all significantly associated for both interventional and observational types of research (p<0.001 for all comparisons). Logistic regression models were created for each ROB domain that included funder, year, and study design. An exploratory analysis with source added to the model, provided little additional information and was not used in the final predictive model.

**Fig 6:**
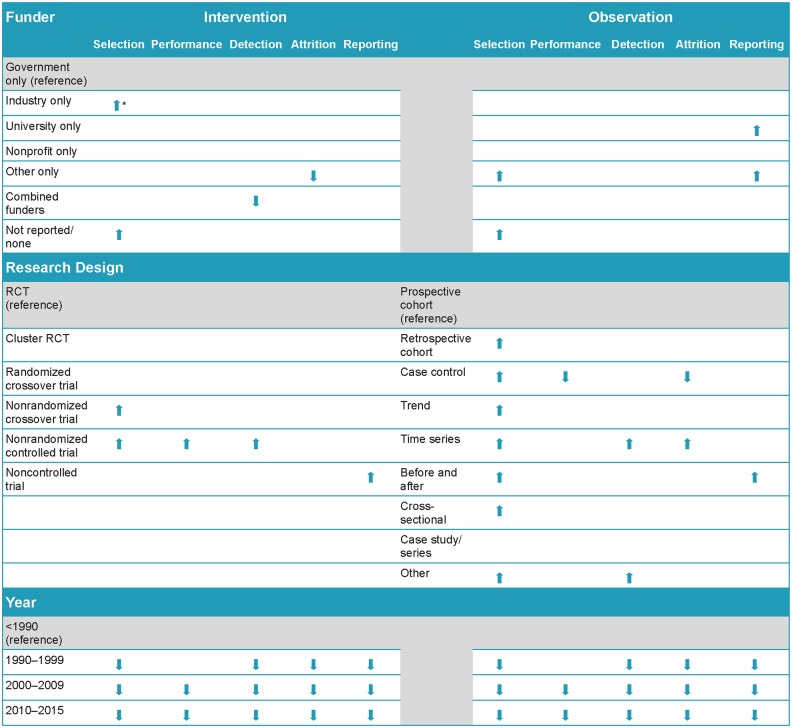
Significant relationships between risk of bias* and funder, research design, and year by research type. *Arrows indicate statistically significant differences from the reference category (p < .0005, Bonferroni correction applied). Downward arrows indicate a lower risk of bias and an upward arrow represents a higher risk of bias.

These final models with funder, year, and study design are reported separately for interventional designs (Table 3) and observational designs (Table 4) and are summarized together in Fig 6.

**Table 3:** Final models for predicting ROB Being met in studies with interventional designs by funder and research type and design: separate models for ROB domains.

Variables	Selection (n = 2777)		Performance (n = 2684)		Detection (n = 2725)		Attrition (n = 2500)		Reporting (n = 2543)	
	OR (95% CI)	p^a^	OR (95% CI)	p^a^	OR (95% CI)	p^a^	OR (95% CI)	p^a^	OR (95% CI)	p ^a^
Government only (ref)		<0.001		<0.001		0.004		0.023		0.066
Industry only	0.56 (0.43, 0.73)	<0.001	1.35 (1.02, 1.79)	0.037	0.96 (0.70, 1.31)	0.805	1.06 (0.76, 1.47)	0.724	0.96 (0.66, 1.41)	0.848
University only	0.84 (0.65, 1.07)	0.161	0.73 (0.57, 0.94)	0.013	1.14 (0.86, 1.51)	0.351	1.26 (0.93, 1.69)	0.134	1.34 (0.96, 1.87)	0.082
Nonprofit only	0.71 (0.51, 0.99)	0.042	1.13 (0.81, 1.59)	0.467	1.03 (0.70, 1.52)	0.874	1.00 (0.67, 1.48)	0.991	1.13 (0.69, 1.86)	0.620
Other only	0.94 (0.62, 1.42)	0.774	1.23 (0.81, 1.87)	0.333	0.91 (0.57, 1.45)	0.700	2.44 (1.32, 4.52)	0.004	0.69 (0.41, 1.16)	0.158
Combined funders	0.92 (0.73, 1.15)	0.444	1.35 (1.08, 1.70)	0.010	1.46 (1.12, 1.91)	0.005	1.41 (1.07, 1.84)	0.013	1.21 (0.90, 1.64)	0.201
Not reported or no funding	0.62 (0.48, 0.80)	<0.001	0.90 (0.70, 1.18)	0.454	0.80 (0.60, 1.06)	0.120	1.04 (0.77, 1.41)	0.779	0.82 (0.58, 1.15)	0.248
<1990 (ref)		<0.001		<0.001		<0.001		<0.001		<0.001
1990–1999	1.56 (1.23, 1.97)	<0.001	1.28 (1.01, 1.63)	0.042	3.01 (2.31, 3.92)	<0.001	2.17 (1.66, 2.85)	<0.001	4.40 (3.25, 5.96)	<0.001
2000–2009	2.27 (1.88, 2.74)	<0.001	1.68 (1.39, 2.02)	<0.001	3.96 (3.21, 4.88)	<0.001	3.54 (2.86, 4.39)	<0.001	5.97 (4.69, 7.60)	<0.001
2010–2015	2.56 (1.96, 3.34)	<0.001	1.77 (1.36, 2.31)	<0.001	5.22 (3.77, 7.24)	<0.001	3.29 (2.36, 4.58)	<0.001	16.53 (9.84, 27.75)	<0.001
RCT (ref)		<0.001		<0.001		<0.001		0.002		0.009
Cluster RCT	0.94 (0.51, 1.75)	0.849	0.90 (0.49, 1.66)	0.733	0.98 (0.46, 2.07)	0.950	1.91 (0.74, 4.92)	0.182	5.97 (0.80, 44.40)	0.081
Randomized crossover trial	1.81 (0.57, 5.74)	0.311	0.90 (0.32, 2.51)	0.843	1.24 (0.34, 4.51)	0.743	0.81 (0.25, 2.64)	0.723	0.42 (0.14, 1.31)	0.137
Nonrandomized crossover trial	0.52 (0.42, 0.63)	<0.001	1.20 (0.97, 1.48)	0.088	0.82 (0.65, 1.04)	0.109	1.48 (1.12, 1.95)	0.006	0.80 (0.61, 1.06)	0.116
Nonrandomized controlled trial	0.34 (0.27, 0.43)	<0.001	0.58 (0.46, 0.73)	<0.001	0.49 (0.38, 0.63)	<0.001	0.73 (0.56, 0.95)	0.021	0.86 (0.63, 1.19)	0.363
Noncontrolled trial	0.51 (0.24, 1.10)	0.084	0.93 (0.42, 2.06)	0.859	0.76 (0.30, 1.92)	0.564	0.65 (0.25, 1.66)	0.365	0.28 (0.11, 0.67)	0.004

CI, confidence interval; OR, odds ratio; RCT, randomized controlled trial; ROB, risk of bias.

^a^p<0.005 was considered statistically significant.

**Table 4:** Final models for predicting ROB being met in studies with observational designs by funder and research type and design: separate models for ROB domains.

Variable	Selection (n = 2727)		Performance (n = 2722)		Detection (n = 2727)		Attrition (n = 2244)		Reporting (n = 2464)	
	OR (95% CI)	p^a^	OR (95% CI)	p^a^	OR (95% CI)	p^a^	OR (95% CI)	p ^a^	OR (95% CI)	p ^a^
Government only (ref)		<0.001		0.805		0.047		0.610		<0.001
Industry only	1.03 (0.62, 1.71)	0.914	1.13 (0.69, 1.85)	0.630	0.78 (0.46, 1.33)	0.367	0.76 (0.41, 1.42)	0.391	0.90 (0.43, 1.89)	0.789
University only	0.88 (0.69, 1.13)	0.315	1.07 (0.84, 1.36)	0.588	0.79 (0.61, 1.03)	0.079	0.79 (0.57, 1.10)	0.165	0.57 (0.41, 0.80)	0.001
Nonprofit only	1.01 (0.68, 1.50)	0.964	0.85 (0.59, 1.24)	0.403	0.99 (0.64, 1.53)	0.971	0.79 (0.47, 1.33)	0.375	1.09 (0.56, 2.11)	0.805
Other only	0.49 (0.34, 0.71)	<0.001	1.05 (0.72, 1.51)	0.816	0.65 (0.44, 0.96)	0.029	0.69 (0.43, 1.13)	0.140	0.36 (0.22, 0.58)	<0.001
Combined funders	0.97 (0.78, 1.21)	0.808	0.91 (0.74, 1.11)	0.352	1.03 (0.81, 1.30)	0.821	0.96 (0.72, 1.28)	0.792	0.92 (0.66, 1.27)	0.599
Not reported or no funding	0.59 (0.46, 0.76)	<0.001	1.03 (0.80, 1.32)	0.843	0.72 (0.55, 0.94)	0.016	0.82 (0.58, 1.16)	0.268	0.61 (0.42, 0.87)	0.006
<1990 (ref)		<0.001		<0.001		<0.001		<0.001		<0.001
1990–1999	2.08 (1.61, 2.70)	<0.001	1.04 (0.81, 1.33)	0.769	3.23 (2.45, 4.26)	<0.001	3.07 (2.23, 4.22)	<0.001	4.30 (3.09, 5.98)	<0.001
2000–2009	2.80 (2.33, 3.36)	<0.001	1.48 (1.24, 1.75)	<0.001	3.74 (3.07, 4.55)	<0.001	5.10 (4.05, 6.44)	<0.001	9.84 (7.52, 12.87)	<0.001
2010–2015	4.83 (3.67, 6.35)	<0.001	1.76 (1.39, 2.23)	<0.001	5.10 (3.81, 6.81)	<0.001	4.74 (3.32, 6.77)	<0.001	42.33 (19.02, 94.22)	<0.001
Prospective cohort (ref)		<0.001		<0.001		<0.001		<0.001		<0.001
Case control	0.34 (0.26, 0.45)	<0.001	1.87 (1.39, 2.51)	<0.001	0.74 (0.54, 1.00)	0.048	2.68 (1.66, 4.32)	<0.001	0.66 (0.45, 0.97)	0.033
Retrospective cohort	0.60 (0.42, 0.84)	<0.003	0.82 (0.59, 1.14)	0.233	0.94 (0.64, 1.37)	0.737	1.33 (0.80, 2.21)	0.279	0.94 (0.58, 1.53)	0.804
Trend study	0.20 (0.10, 0.42)	<0.001	1.93 (0.89, 4.20)	0.095	0.71 (0.33, 1.53)	0.385	0.69 (0.29, 1.66)	0.409	2.06 (0.48, 8.79)	0.329
Cross-sectional study	0.60 (0.50, 0.74)	<0.001	1.02 (0.84, 1.23)	0.837	0.82 (0.66, 1.02)	0.070	1.11 (0.86, 1.43)	0.431	1.21 (0.90, 1.62)	0.200
Case study or case series	1.09 (0.55, 2.16)	0.807	1.41 (0.72, 2.75)	0.315	0.86 (0.43, 1.74)	0.684	1.27 (0.51, 3.18)	0.610	0.40 (0.20, 0.83)	0.013
Before-after study	0.31 (0.21, 0.47)	<0.001	0.69 (0.47, 1.01)	0.056	0.70 (0.46, 1.07)	0.096	0.68 (0.42, 1.09)	0.108	0.42 (0.26, 0.68)	<0.001
Time series	0.34 (0.22, 0.55)	<0.001	0.60 (0.38, 0.94)	0.025	0.51 (0.32, 0.81)	0.005	0.45 (0.27, 0.75)	0.002	0.56 (0.33, 0.95)	0.032
Other descriptive	0.19 (0.11, 0.33)	<0.001	1.01 (0.61, 1.69)	0.967	0.27 (0.16, 0.46)	<0.001	1.24 (0.56, 2.73)	0.600	0.56 (0.30, 1.06)	0.077

CI, confidence interval; OR, odds ratio; ROB, risk of bias.

^a^p<0.005 was considered statistically significant.

#### Intervention studies and ROB

Across domains, category of publication year predicted lower risk of bias in more recent published interventional research (Table 3). With the exception of the Performance domain in 1990 to 1999 year category, all domains were significantly more likely to have lower risk of bias in more recent years when compared to research published prior to 1990. The greatest improvements were shown for the Reporting domain in the research published between 2010 to 2015 (OR = 16.53). Thus, even though there is a clear secular trend for studies published since 2000 to be at lower ROB, the degree of improvements compared to the <1990 period are not even across domains.

There was no clear pattern regarding the ability funding source to predict risk of bias in interventional research. Compared to government-only funding, differences among funders were identified in only three domains: Selection, Detection and Attrition for Interventional research. Within the Selection domain, industry-only funding was associated with a 44% increase in the likelihood of having a higher risk of bias, while funding not reported or no funding was associated with a 38% increase in the likelihood of a higher risk of bias (p<0.001 for both) compared to government-only funding. In the Detection domain, studies that had combined funding (multiple sources) were 46% more likely to achieve a lower risk of bias (better than government-only funding). Other-only source of funding was significant; however, since this included diverse funders, it is of limited usefulness in drawing conclusions about potential areas of improvement. All other domains and sources of funding did not predict differences in risk of bias when compared to government funding.

The only clear pattern for specific study design across domains in interventional research was that, compared to RCTs, nonrandomized controlled trials were more likely to be rated with a high risk of bias (ORs varied from 0.34. to 0.86), though these lower ORs were statistically significant only for the Selection, Performance and Detection domains. Findings for specific research designs (e.g., cluster RCT, randomized crossover trials, and noncontrolled trials) should be interpreted with caution due to the small sample sizes which inevitably led to a loss of power for these comparisons. Though the estimates were statistically significant only for increased risk of bias within the Reporting domain (OR = 0.28) for the noncontrolled trials, there was a nonsignificant trend across other domains.

#### Observational *s*tudies and ROB

Similar to the results from interventional studies, with one exception, the Attrition domain, category of publication year predicted lower risk of bias in more recent published observational research (Table 4). Again with the exception of the Performance domain in 1990 to 1999 year category, all domains were significantly more likely to have lower risk of bias in more recent years when compared to research published prior to 1990. The greatest improvements was again shown for the Reporting domain in the research published between 2010 to 2015 (OR = 42.33). The degree of improvements over the <1990 period is not even across domains.

As with interventional designs, there was no clear pattern of the effect of funder. Indeed, funder made a significant contribution to the models only for Selection and Reporting in observational research (p<0.001 for both). For Selection, other-only and funding not reported or no funding were both less likely to meet ROB criteria (i.e., more likely to be at higher risk of bias) than government-only funded studies (OR = 0.49 and 0.59, respectively). In the Reporting domain, other-only and university-only funding was more likely to be at higher risk of bias than government-only funded studies (OR = 0.36 and 0.57, respectively).

For study design in observational research, there was no clear pattern across domains when various observational designs were compared to prospective cohort designs. While the effect of study design was significant across domains (p<0.001 for all), the effects relative to prospective cohort designs was inconsistent. For instance, in the Selection domain, all observational designs except for case study or case series were significantly more likely to be at higher risk of bias than the prospective cohort studies. In contrast, differences were limited in other domains. For instance, case control designs were significantly more likely to have lower bias compared to prospective cohort designs in both Performance and Attrition domains (OR = 1.87 and 2.68, respectively); however, time series designs were significantly more likely to have higher bias compared to prospective cohort deigns in both Detection and Attrition domains (OR = 0.51 and 0.45, respectively).

Based on our analysis we have robust estimates of predictors of risk of bias. The results are summarized in Fig 6.

## Discussion

This study examined the degree to which ROB domains are met to identify where shortfalls exist in the current body of food- and nutrition-related research drawn from three large organizations conducting systematic reviews. This work’s unique contribution is the breadth of topics addressed and its use of the ROB domains as a way to characterize where food and nutrition research may be most improved. Strengths include the large sample size, diversity of topics (e.g., physical activity, food safety, dietary supplement, telenutrition, nutrition counseling), diversity of funding sources, different purpose of the systematic reviews (e.g., public health and policy approach of the NEL, clinical practice recommendations and nutrition therapy approach of the EAL, and association between nutrients and health and disease markers approach of the AHRQ), inclusion of both intervention and observation research, breadth of research designs, and consistent use of the same instruments for a large proportion of the research sample. Using appraisal records from the three data sources provides a broad representation of food- and nutrition-related studies, covering a range of current topics applicable to nutrition policy and practice. Sample distributions of funders from industry, university nonprofit and other were similar to other estimates of funding for life sciences [26]. However, Lanahan reported higher levels of government funding in life sciences (61%) versus our sample with 23%. Lanahan did not include a separate category for combined funding. In our research the combined funder category (25.6%) also included studies with government funding plus other funding. However, even if these two categories were added together, the body of research being used in systematic reviews from these three data sources has less government funding than life sciences in general. Limitations of this research include the need to map individual questions used in critical appraisal instruments to ROB domains, use of ratings from more than one critical appraisal instrument, imperfect tools for critical appraisals, use of subjective ratings by different analysts, purposive sampling of the full body of food and nutrition research, and inability to account for other potential confounders affecting the use of the critical appraisal instruments or data source (e.g., variation in training of analysts, dates of systematic review projects, diversity of topics, organizational resources available, and analysis by topic, such as human versus non-human studies). Since the comparison of ratings of the same study when evaluated more than once showed very high agreement, and the domains that predicted overall quality between the two data sources were quite consistent, the data source was not helpful in our analysis and did not contribute to the identification of areas for improvement in the food and nutrition body of original research. Factors specific to the data sources could be a topic for future research focusing on systematic review methodology and may identify opportunities for improvement in systematic reviews. Future research might also focus on the impact of changing emphasis on full reporting of funding support over time and analysis at the individual critical appraisal item within each ROB domain.

The three ROB domains that provide the greatest opportunity for improvement are the Selection, Performance, and Detection domains. These were the most often not met and were consistently the significant predictors of lower overall quality ratings (higher risk of bias) in both interventional and observational research.

### Selection ROB domain

Intervention research and observational research studies met the Selection ROB domain criteria 56% and 60%, respectively. As anticipated, nonrandomized crossover and nonrandomized clinical trials were significantly less likely to meet the Selection ROB criteria/higher risk of bias than the RCT reference standard. In our sample of food and nutrition research, 32% of the intervention research reports were nonrandomized trials. Use of these nonrandomized research designs should be limited to specific circumstances where randomization is not possible and group differences can be minimized, identified, and adjusted for. Within the observational research type, all of the research designs except case control were significantly less likely to meet Selection domain criteria/higher risk of bias when compared to prospective cohort research. Using enhanced methodologies for eligibility criteria, identifying groups appropriately, and adjusting for group differences within the observational research designs may be able to enhance the ability to meet Selection domain criteria.

### Detection ROB domain

Detection domain criteria were met by 77% of the intervention research articles and 78% of the observation research articles; only nonrandomized controlled trials were significantly less likely to meet these criteria/higher risk of bias than RCT intervention research designs. In observational designs, time series, cross-sectional, and other descriptive studies were less likely to meet the Detection ROB domain criteria/higher risk of bias than prospective cohort. Improvements in determining the sample size and power, accurate measurement of outcomes and harms, sufficient length of follow-up, and statistical adjustments for confounders specific to food- and nutrition-related research are opportunities for reducing the risk of detection bias in all research designs.

### Performance ROB domain

Performance ROB domain criteria were met by 62% of all intervention research studies and 59% of observational research studies. Only the nonrandomized controlled trial interventional design was likely to have higher risk of bias for the Performance domain than the comparator, RCTs. This domain did not start improving over time until 2000 and then the improvements were smaller than other domains. Either the critical appraisal instruments are not sensitive to food and nutrition research specific aspects of performance and able to capture improvements or Performance has not truly improved as much over time. Opportunities for enhancements in methods related to intervention delivery and measurement of interventions or exposures that address challenges specific to food- and nutrition-related research regardless of research design should be systematically identified.

### Implications of research design

The ROB domain criteria findings relate to the hierarchy of evidence and reflect the inherent limitations of specific research designs [13, 27]. One implication is that the quality of the body of food- and nutrition-related research will improve when there are shifts from research designs lower in the hierarchy of evidence to those higher in the hierarchy.

However, it is clearly acknowledged that different systematic review questions require different types of research designs to inform the answers. For example, questions related to diagnosis are not answered by RCTs because they are more appropriately answered by using cross-sectional studies, prognosis questions are answered using prospective (inception) cohort research, and questions about harms are answered using nested case control studies [28]. This sample encompasses many different types of research designs because the questions asked vary. For example, an AHRQ report might address a question about the efficacy of omega-3 fatty acids to improve respiratory outcomes among individuals with asthma. An NEL topic might address the relationship between neighborhood and community access to food retail settings and individuals’ dietary intake and quality or the relationship between dietary patterns and risk of breast cancer. EAL topics may range from about questions about the long-term effectiveness of following a gluten-free dietary pattern on gastrointestinal symptoms for people with celiac disease to the food safety behaviors of adults related to microwave cooking.

The relationships between the specific research design and the comparator (RCTs for intervention and prospective cohort for observational research) should also be noted. However interpretation of results should be moderated by small sample sizes in some research designs (e.g., noncontrolled trial, randomized crossover trial trend study, and case study/or case series). Intervention designs are more homogeneous in that they are all exploring a cause-and-effect relationship; however, each of the observational designs is different in nature and takes different approaches to describe phenomena or relationships. It is not surprising that more significant relationships were found when other observational designs were compared to the prospective cohort design.

### Implications of reported improvements by year

In this study we found that publication year was positively associated with the likelihood of meeting all ROB domains with the exception of the Performance domain. Significant improvement in the Performance domain only started after 2000 when compared to the reference category (<1990). Whether this is related to increasing emphasis on guidelines to enhance reporting or whether the research methodology has actually improved is unknown. Various design-specific guidelines have been published, beginning in 1999 with Consolidated Standards of Reporting Trials (CONSORT); however, research on the implementation and impact of research publishing guidelines has shown mixed results [29–32].

### Implications of relationship of source of funding

There is a strong desire to enhance the integrity of food- and nutrition-related research. Often, the focus has been on source of funding as a significant way in which bias is introduced into research. While previous research has called this assumption into question, it is still hotly debated [1, 33–37]. The emphasis from peer-reviewed journals on full funding disclosure also has changed over time. Although our sample included three different systematic review sources, the predominant contributor was EAL. Therefore, it is not surprising that overall results regarding funding are similar to the previous study on funding and research quality [1]. The “not reported/none” type was a significant predictor of higher risk of bias for Selection in both interventional and observational and “other funder” type was a significant predictor of a higher risk of bias for Selection and Reporting for observational research. However, it is unclear whether authors reported support for their time through salary as “funding.” University-only funding was a significant predictor higher risk of bias for Reporting in observational research, while industry-only funding was a significant predictor for higher risk of bias for Selection in interventional research. A recent Cochrane review found that industry-funded research reported one of the factors (satisfactory blinding; related to Performance and Detection ROB criteria) more often than nonindustry-funded research [18].

An updated analysis that also evaluated direction of findings concluded that while industry-funded research is more likely to have favorable results, no systematic bias had been identified using standard ROB criteria [18, 38]. Usually if the ROB criteria are met, then there would normally be a high level of confidence that results are likely to be unbiased and can be trusted and replicated. However, if more favorable findings in industry-funded research are not a function of methodological rigor (at least as indicated in the ROB domains of existing assessment tools), then other explanations for the disproportionate amount of favorable conclusions need further investigation. Chartres et al. completed a similar review specifically on food industry-funded research, and they reported that the association between favorable conclusions and industry funding did not reach the threshold for significance and evidence was insufficient to indicate that the quality of the research itself was impacted [39]. Funding source may bias research more generally, but not largely via methodological rigor.

These findings indicate that receiving industry funding is not consistently associated with producing research results that are considered “biased” using the standard ROB criteria that reflect the rigor of the research. The one exception was a relationship in intervention research for only one of the five ROB domains, Selection. This coupled with the finding that combined funders were less likely to have risk of bias for detection, lend support for the recent dialogue about the importance of identifying and adhering to principles that guide industry- or public-private partnership–funded research to ensure that the research is conducted to the highest rigor possible without undue influence on the findings or conclusions [40–45]. The dialogue may need to shift from a discussion of how the research was conducted (e.g., Were the results of this research study biased due to methodological limitations?) to what topics are funded for the body of research (e.g., What questions are unanswered, specifically related to harms or lack of effectiveness?) and whether negative study results are published.

### Implications for the food and nutrition research enterprise

Since standard ROB tools are not detecting bias related to direction of findings in food and nutrition research, this may suggest that future research should identify factors leading to this phenomenon and perhaps publication guidelines that include those factors should be developed. It may be that the factors are not related to the individual research study and other systems such as research registries need to be developed to monitor these factors (e.g., funding practices, publishing practices).

The Strengthening the Reporting of Observational studies in Epidemiology (STROBE) Statement currently has a nutrition extension that includes 24 specific nutrition items on their checklist called STROBE-nut [46]. This has been elaborated in an article explaining the application of each of the items [47]. It is noteworthy that there are currently 11 different extensions to the CONSORT Guideline, including Herbal Medicinal Interventions (2006) and Non-Pharmacologic Treatment Interventions (2007) [48–51]. However, there currently is no extension that provides guidance for food- and nutrition-related studies. For example, the importance of characterizing the nutritional supplement or the background dietary or environmental intake may not be adequately described in the published research and may therefore not be captured in the application of the ROB evaluation [52, 53]. Having publication guidelines that require the researcher to address this issue more fully will allow for a more complete evaluation of the Performance and Detection ROB domain criteria.

A Cochrane review used a four tier-approach to identify ways to measure impact of educational interventions focused on improving research integrity [54]. This may provide a useful framework for considering what potential future activities could lead to improvements within food- and nutrition-related research. The four primary outcomes from research integrity educational interventions were as follows: acquisition of knowledge and/or skills, modification of attitudes and/or perceptions, organizational change attributable to the education, and behavioral change as either intention to change or actual change in research behavior [54–56]. Implications for the research enterprise can be addressed in academia, professional societies, funding agencies, organizations conducting systematic reviews, and journals in a variety of ways, either through implementing policy and procedures or providing information and education about the use of ROB ratings in systematic reviews and ways to strengthen their original research designs to address selection, performance, and detection in design, statistical analysis, and reporting. This research sets the stage for beginning a dialogue among all stakeholders of food- and nutrition-related research about to activities, policies, and procedures that could address the shortcomings identified in this research.

## Conclusions

Overall, the greatest opportunity for improvement in food- and nutrition-related research as reflected by this sample is in the three domains of Selection, Performance, and Detection. Failure to meet these three ROB domain criteria greatly increased the likelihood of a neutral or negative quality rating.

Over time, the percentage of published research that meets the ROB domain criteria (lower risk of bias) has steadily increased with the exception of Performance, which only began improvement in 2000. There were a few instances in which non-government-only funding was a significant predictor of whether there was a higher risk of bias for Selection when compared to government funding and there were also a few instances where non-government-only funding predicted less risk of bias for Detection and Attrition. However, research design and publication year were more consistent predictors of ROB domain ratings in food and nutrition research.

There was surprisingly little overlap (10%) and very high consistency in the ROB domain ratings for the research articles with more than one critical appraisal record. The largest overlap was between the EAL and NEL, where there was extremely high agreement in the ROB domain ratings.

These results support focusing on the three ROB domains that can be strengthened, and they allow the food and nutrition research community to focus on constructive ways to improve the overall body of research rather than investigate source of funding as the primary predictor of ROB. This research sets the stage for establishing initiatives to support or create research environments that enhance rigor of research and demand research integrity.

## Supporting information

S1 Table Cross-tabulation of funding source by study design. (DOCX)

S2 Table Proportion of studies meeting the ROB criteria by research type and design. (DOCX)

S1 Fig Flow diagram of critical appraisal record selection. (DOCX)

S2 Fig Example of a search plan from the Academy Evidence Analysis Library. (DOCX)

S3 Fig Example of a search strategy and flow from an AHRQ report. (PDF)
