# Supplementary material for: Using risk of bias domains to identify opportunities for improvement in food- and nutrition-related research: An evaluation of research type and design, year of publication, and source of funding
Source: PLoS One. 2018 Jul 5;13(7):e0197425. doi: 10.1371/journal.pone.0197425 (PMC6033375; doi:10.1371/journal.pone.0197425)
Supplement: S1 Table — (DOCX) [file pone.0197425.s001.docx]

**S1 Table. Crosstabulation of Funding Source by Study Design.**

| **Design** |  | **Funder** | | | | | | | **Total** |
| --- | --- | --- | --- | --- | --- | --- | --- | --- | --- |
|  |  | **Government Only** | **Industry Only** | **University Only** | **Nonprofit Only** | **Other Only** | **Combined Funders** | **Not Reported or No Funding** |  |
| **Interventional Designs** | | |  |  |  |  |  |  |  |
| Noncontrolled trial | n | 9 | 1 | 4 | 1 | 5 | 7 | 1 | 28 |
|  | % | 1.7 | 0.3 | 0.8 | 0.5 | 4.1 | 0.9 | 0.2 | 1.0 |
| Nonrandomized controlled trial | n | 65 | 31 | 104 | 22 | 16 | 80 | 64 | 382 |
|  | % | 12.6 | 8.1 | 21.8 | 10.8 | 13.2 | 10.5 | 14.3 | 13.2 |
| Nonrandomized crossover trial | n | 63 | 82 | 109 | 33 | 22 | 168 | 63 | 540 |
|  | % | 12.2 | 21.5 | 22.9 | 16.3 | 18.2 | 22.1 | 14.1 | 18.6 |
| RCT | n | 359 | 259 | 258 | 135 | 75 | 489 | 313 | 1888 |
|  | % | 69.7 | 67.8 | 54.1 | 66.5 | 62.0 | 64.4 | 70.0 | 65.0 |
| Cluster RCT | n | 11 | 6 | 2 | 10 | 2 | 9 | 5 | 45 |
|  | % | 2.1 | 1.6 | 0.4 | 4.9 | 1.7 | 1.2 | 1.1 | 1.5 |
| Randomized crossover trial | n | 8 | 3 | 0 | 2 | 1 | 6 | 1 | 21 |
|  | % | 1.6 | 0.8 | 0.0 | 1.0 | 0.8 | 0.8 | 0.2 | 0.7 |
| Total | n | 515 | 382 | 477 | 203 | 121 | 759 | 447 | 2904 |
|  | % | 100.0 | 100.0 | 100.0 | 100.0 | 100.0 | 100.0 | 100.0 | 100.0 |
| **Observational Designs** | | |  |  |  |  |  |  |  |
| Other descriptive | n | 16 | 2 | 11 | 2 | 5 | 14 | 15 | 65 |
|  | % | 1.9 | 2.5 | 2.3 | 1.5 | 3.5 | 2.0 | 3.8 | 2.3 |
| Case study or case series | n | 7 | 0 | 14 | 2 | 2 | 7 | 10 | 42 |
|  | % | 0.8 | 0.0 | 2.9 | 1.5 | 1.4 | 1.0 | 2.5 | 1.5 |
| Cross-sectional study | n | 251 | 32 | 216 | 38 | 61 | 221 | 120 | 939 |
|  | % | 29.9 | 40.5 | 44.9 | 28.4 | 42.7 | 31.8 | 30.2 | 33.9 |
| Trend study | n | 17 | 0 | 1 | 1 | 0 | 5 | 10 | 34 |
|  | % | 2.0 | 0.0 | 0.2 | 0.7 | 0.0 | 0.7 | 2.5 | 1.2 |
| Retrospective cohort study | n | 28 | 4 | 54 | 6 | 14 | 34 | 37 | 177 |
|  | % | 3.3 | 5.1 | 11.2 | 4.5 | 9.8 | 4.9 | 9.3 | 6.4 |
| Case control study | n | 79 | 6 | 59 | 25 | 14 | 54 | 53 | 290 |
|  | % | 9.4 | 7.6 | 12.3 | 18.7 | 9.8 | 7.8 | 13.3 | 10.5 |
| Prospective cohort | n | 407 | 25 | 80 | 54 | 31 | 304 | 115 | 1016 |
|  | % | 48.5 | 31.6 | 16.6 | 40.3 | 21.7 | 43.7 | 28.9 | 36.7 |
| Before-after study | n | 23 | 3 | 31 | 5 | 12 | 30 | 17 | 121 |
|  | % | 2.7 | 3.8 | 6.4 | 3.7 | 8.4 | 4.3 | 4.3 | 4.4 |
| Time series | n | 12 | 7 | 15 | 1 | 4 | 27 | 21 | 87 |
|  | % | 1.4 | 8.9 | 3.1 | 0.7 | 2.8 | 3.9 | 5.3 | 3.1 |
| Total | n | 840 | 79 | 481 | 134 | 143 | 696 | 398 | 2771 |
|  | % | 100.0 | 100.0 | 100.0 | 100.0 | 100.0 | 100.0 | 100.0 | 100.0 |
