# Supplementary material for: Using risk of bias domains to identify opportunities for improvement in food- and nutrition-related research: An evaluation of research type and design, year of publication, and source of funding
Source: PLoS One. 2018 Jul 5;13(7):e0197425. doi: 10.1371/journal.pone.0197425 (PMC6033375; doi:10.1371/journal.pone.0197425)
Supplement: S2 Table — (DOCX) [file pone.0197425.s002.docx]

**S2 Table. Proportion of Studies Meeting the ROB Criteria by Research Type and Design.**

| **Type and Design** |  | **Selection** | | **Performance** | | **Detection** | | **Attrition** | | **Other ROB** | |
| --- | --- | --- | --- | --- | --- | --- | --- | --- | --- | --- | --- |
|  |  | **Criteria met** | **Total** | **Criteria met** | **Total** | **Criteria met** | **Total** | **Criteria met** | **Total** | **Criteria met** | **Total** |
| **Clinical trials** |  |  |  |  |  |  |  |  |  |  |  |
| RCT | n | 1139 | 1795 | 1081 | 1705 | 1403 | 1743 | 1273 | 1625 | 1413 | 1633 |
|  | % | 63.5 |  | 63.4 |  | 80.5 |  | 78.3 |  | 86.5 |  |
| Cluster RCT | n | 28 | 45 | 28 | 45 | 36 | 45 | 36 | 41 | 38 | 39 |
|  | % | 62.2 |  | 62.2 |  | 80.0 |  | 87.8 |  | 97.4 |  |
| Randomized crossover trial | n | 12 | 16 | 10 | 16 | 13 | 16 | 11 | 15 | 10 | 15 |
|  | % | 75.0 |  | 62.5 |  | 81.3 |  | 73.3 |  | 66.7 |  |
| Nonrandomized crossover trial | n | 237 | 521 | 348 | 520 | 395 | 521 | 375 | 451 | 411 | 501 |
|  | % | 45.5^a^ |  | 66.9 |  | 75.8 |  | 83.1 |  | 82.0 |  |
| Nonrandomized controlled trial | n | 133 | 373 | 175 | 371 | 238 | 373 | 244 | 344 | 270 | 330 |
|  | % | 35.7^a^ |  | 47.2^a^ |  | 63.8 |  | 70.9 |  | 81.8 |  |
| Noncontrolled trial | n | 14 | 27 | 17 | 27 | 21 | 27 | 18 | 24 | 17 | 25 |
|  | % | 51.9 |  | 63.0 |  | 77.8 |  | 75.0 |  | 68.0 |  |
| Total | n | 1563 | 2777 | 1659 | 2684 | 2106 | 2725 | 1957 | 2500 | 2159 | 2543 |
|  | % | 56.3 |  | 61.8 |  | 77.3 |  | 78.3 |  | 84.9 |  |
| **Observational designs** |  |  |  |  |  |  |  |  |  |  |  |
| Prospective cohort | n | 740 | 997 | 595 | 994 | 793 | 997 | 694 | 844 | 722 | 805 |
|  | % | 74.2 |  | 59.9 |  | 79.5 |  | 82.2 |  | 89.7 |  |
| Retrospective cohort study | n | 102 | 176 | 94 | 176 | 131 | 176 | 100 | 121 | 142 | 168 |
|  | % | 58.0 |  | 53.4 |  | 74.4 |  | 82.6 |  | 84.5 |  |
| Case control study | n | 125 | 279 | 200 | 278 | 197 | 279 | 217 | 239 | 198 | 251 |
|  | % | 44.8^a^ |  | 71.9 |  | 70.6 |  | 90.8 |  | 78.9 |  |
| Trend study | n | 11 | 34 | 25 | 34 | 24 | 34 | 22 | 29 | 32 | 34 |
|  | % | 32.4^a^ |  | 73.5 |  | 70.6 |  | 75.9 |  | 94.1 |  |
| Time series | n | 35 | 87 | 38 | 87 | 50 | 87 | 46 | 78 | 60 | 86 |
|  | % | 40.2^a^ |  | 43.7^a^ |  | 57.5 |  | 59.0 |  | 69.8 |  |
| Before-after study | n | 50 | 119 | 58 | 119 | 81 | 119 | 71 | 99 | 84 | 118 |
|  | % | 42.0^a^ |  | 48.7^a^ |  | 68.1 |  | 71.7 |  | 71.2 |  |
| Cross-sectional study | n | 536 | 929 | 542 | 929 | 668 | 929 | 609 | 756 | 779 | 897 |
|  | % | 57.7 |  | 58.3 |  | 71.9 |  | 80.6 |  | 86.8 |  |
| Case study or case series | n | 28 | 41 | 26 | 40 | 29 | 41 | 26 | 32 | 26 | 40 |
|  | % | 68.3 |  | 65.0 |  | 70.7 |  | 81.3 |  | 65.0 |  |
| Other descriptive | n | 19 | 65 | 38 | 65 | 29 | 65 | 38 | 46 | 49 | 65 |
|  | % | 29.2^a^ |  | 58.5 |  | 44.6^a^ |  | 82.6 |  | 75.4 |  |
| Total | n | 1646 | 2727 | 1616 | 2722 | 2002 | 2727 | 1823 | 2244 | 2092 | 2464 |
|  | % | 60.4 |  | 59.4 |  | 73.4 |  | 81.2 |  | 84.9 |  |

There was a significant association (p<0.001) between study design and whether the ROB criteria were met for all domains (chi-square). ROB, risk of bias.

^a^Cells indicate instances where less than 50% of the articles included in the sample met the ROB criteria.
