# Supplementary material for: Using risk of bias domains to identify opportunities for improvement in food- and nutrition-related research: An evaluation of research type and design, year of publication, and source of funding
Source: PLoS One. 2018 Jul 5;13(7):e0197425. doi: 10.1371/journal.pone.0197425 (PMC6033375; doi:10.1371/journal.pone.0197425)
Supplement: S1 Fig — (DOCX) [file pone.0197425.s003.docx]

**S-1 Figure. Flow Diagram of Critical Appraisal Record Selection***

Projects identified

**Nutrition Evidence Library**

[NEL Website](https://www.cnpp.usda.gov/nutritionevidencelibrary)

N = 4 projects

**AHRQ Evidence Based Reports**

[AHRQ Website](https://www.ahrq.gov/research/findings/evidence-based-reports/index.html),)

n=488 AHRQ reports

**Evidence Analysis Library**

[EAL Website](https://www.andeal.org/default.cfm)

N= 42 projects

Projects and critical appraisal records included

468 projects excluded

Reports screened to be Food and Nutrition Related*

*Data selection date – Reviews posted on websites in August 2016

**Food and nutrition-related systematic reviews are topics that impact decisions about nutrition needs or nutrition outcomes for humans. Systematic review topics considered to be "nutrition-related" could be from any of the realms of research used by dietitians, e.g. Nutrition research, food science, behavioral and social sciences, management, basic sciences or dietetics with the focus on determining nutrition needs, nutrition care or nutrition outcomes. All EAL and NEL were food and nutrition related but AHRQ reports included many other topics e.g. medical devises, pharmacological or medical treatment.

***Critical appraisal records for a single article are included in more than one system or more than one time in AHRQ reports. Which ROB domains were rated depended on study design. Some critical appraisals have ROB ratings only, some have overall quality only, and some have both.

****Data at data repository. Unduplicated (one critical appraisal record for one article, differences reconciled as per methodology)

Critical appraisal records with **both** overall quality and individual item ratings across all 3 systems

n=3879

Critical appraisal records with ROB ratings across all 3 systems

n=5504 Selection n=5452 Detection n=5007 Reporting

n=5406 Performance n=4744 Attrition

Critical appraisal records with overall quality rating across all 3 systems

n=4760

Critical appraisal records across all 3 systems

n=5675****

Critical appraisal records data without duplicates in final dataset

n= 20 projects

n= 909 critical appraisal records***

All 4 projects

n= 1196 critical appraisal records***

All 42 projects

n= 4220 critical appraisal records***
