# Supplementary material for: Using risk of bias domains to identify opportunities for improvement in food- and nutrition-related research: An evaluation of research type and design, year of publication, and source of funding
Source: PLoS One. 2018 Jul 5;13(7):e0197425. doi: 10.1371/journal.pone.0197425 (PMC6033375; doi:10.1371/journal.pone.0197425)
Supplement: S2 Fig — (DOCX) [file pone.0197425.s004.docx]

**S2 Figure. Example of a search plan from the Academy Evidence Analysis Library.**

Within the Diabetes 1 and 2 Project there are 4 different projects by year and in the 2015 update alone, there are 12 separate search plans on the following topics: Effectiveness of MNT provided by RD/RND, Carbohydrate amount (shown here), Carbohydrate management strategy, Fiber, Glycemic load, FDA approved non-nutritive sweeteners, Nutrition sweeteners, Fat amount, Type of fat, Omega 3 Fatty Acids, Protein amount, and Types of Protein (vegetable vs animal protein)

**Carbohydrate amount**

**Search Plan and Results**

**Evidence Analysis Question**

[In adults with type 1 and type 2 diabetes, what is the relationship of differing amounts of carbohydrate, independent of weight loss, on glycemia (A1C or glucose)?](https://www.andeal.org/conclusion.cfm?conclusion_statement_id=251999)

[In adults with type 1 and type 2 diabetes, what is the relationship of differing amounts of carbohydrate, independent of weight loss, on insulin levels (exogenous or endogenous)?](https://www.andeal.org/conclusion.cfm?conclusion_statement_id=252115)

[In adults with type 1 and type 2 diabetes, what is the relationship of differing amounts of carbohydrate, independent of weight loss, on CVD risk factors (lipids or blood pressure)?](https://www.andeal.org/conclusion.cfm?conclusion_statement_id=252116)

**Date of Literature Review**

February 2013

**Inclusion Criteria**

**Age**

Adults over age 18 years.

**Setting**

Outpatient and ambulatory care.

**Health Status**

Any.

**Nutrition-related Problem/Condition**

Overweight and obesity with and without co-morbid conditions.

**Study Design Preferences**

RCTs (Class A), cohort studies (Class B) and non-randomized clinical trials (Class C) that have a study duration of at least 12 weeks or three months.

**Size of Study Groups**

The sample size must equal 10 individuals for each study group. For example, this would include 10 patients in the intervention group and 10 patients in the control or comparison group.

**Year** **Range**

February 2007 to February 2013.

**Authorship**

- If an author is included on more than one review article or primary research article that is similar in content, the most recent review or article will be accepted and earlier versions will be rejected
- If an author is included on more than one review article or primary research article and the content is different, then both reviews may be accepted.

**Language**

Limited to articles in English.

**Exclusion Criteria**

**Age**

Children and adolescents (under age 18 years).

**Setting**

Inpatient or acute care.

**Health Status**

Patients with a poor prognosis.

**Nutrition Related Problem/Condition**

Critical illness and other disease and conditions.

**Study Design Preferences**

Cross-sectional and before-and-after studies (Class D), and included study designs with durations of less than 12 weeks or three months.

**Study Dropout Rate**

More than 20%.

**Year** **Range**

Prior to February 2007.

**Authorship**

Studies by same author similar in content.

**Language**

Articles not in English.

**Search Terms: Search Vocabulary**

**Health Condition**

Diabet*.

**Intervention**

- Carbohydrate intake
- Dietary carbohydrate.

**Type of Study Design**

- RCTs (Class A)
- Cohort studies (Class B)
- Non-randomized clinical trials (Class C).

**Electronic Databases**

- *Database:* PubMed
- *Search Terms:* (Diabet*) and ("carbohydrate intake" or "dietary carbohydrate")
- *Hits:* 83
- *Articles to review:* 12.

CENTRAL database not used.

Other databases not used.

**Total articles identified to review from electronic databases:** 12

**Articles Identified from other sources:**

**Articles Identified Through: Ajala O, English P, Pinkney J. Systematic review and meta-analysis of different dietary approaches to the management of type 2 diabetes. *Am J Clin Nutr.*2013 Jan 30. [Epub ahead of print.]**

Brehm BJ, Lattin BL, Summer SS, Boback JA, Gilchrist GM, Jandacek RJ, D’Alessio DA. One-year comparison of a high-monounsaturated fat diet with a high-carbohydrate diet in type 2 diabetes. *Diabetes Care*. 2009; 32: 215-220.

Davis NJ, Tomuta N, Schechter C, Isasi CR, Segal-Isaacson CJ, Stein D, Zonszein J, Wylie-Rosett J. Comparative study of the effects of a one-year dietary intervention of a low-carbohydrate diet vs. a low-fat diet on weight and glycemic control in type 2 diabetes. *Diabetes Care*. 2009; 32: 1, 147-1, 152.

Elhayany A, Lustman A, Abel R, Attal-Singer J, Vinker S. A low carbohydrate Mediterranean diet improves cardiovascular risk factors and diabetes control among overweight patients with type 2 diabetes mellitus: A one-year prospective randomized intervention study. *Diabetes Obes Metab.* 2010; 12: 204–209.

Haimoto H, Iwata M, Wakai K, Umegaki H. Long-term effects of a diet loosely restricting carbohydrates on HbA1c levels, BMI and tapering of sulfonylureas in type 2 diabetes: A two-year follow-up study. *Diabetes Res Clin Pract.* 2008; 79(2): 350-356.

Iqbal N, Vetter ML, Moore RH, Chittams JL, Dalton-Bakes CV, Dowd M, Williams-Smith C, Cardillo S, Wadden TA. Effects of a low-intensity intervention that prescribed a low-carbohydrate vs. a low-fat diet in obese. *Obesity (Silver Spring).* 2010; 18: 1, 733-1, 738.

Samaha FF, Iqbal N, Seshadri P, Chicano KL, Daily DA, McGrory J, Williams T, Williams M, Gracely EJ, Stern L. A low-carbohydrate as compared with a low-fat diet in severe obesity. *N Engl J Med*. 2003; 348: 2, 074-2, 081.

Stern L, Iqbal N, Seshadri P, Chicano KL, Daily DA, McGrory J, Williams T, Gracely EJ, Samaha FF. The effects of low-carbohydrate vs. conventional weight loss diets in severely obese adults: one year follow-up of a randomized trial. *Ann Intern Med*. 2004; 140: 778-785.

Westman EC, Yancy WS Jr, Mavropoulos JC, Marquart M, McDuffie JR. The effect of a low-carbohydrate, ketogenic diet vs. a low-glycemic index diet on glycemic control in type 2 diabetes mellitus. *Nutr Metab (Lond)*. 2008; 5: 36.

Wolever TM, Gibbs AL, Mehling C, Chiasson JL, Connelly PW, Josse RG, Leiter LA, Maheux P, Rabasa-Lhoret R, Rodger NW, Ryan EA. The Canadian Trial of Carbohydrates in Diabetes (CCD), a one-year controlled trial of low-glycemic-index dietary carbohydrate in type 2 diabetes: No effect on glycated hemoglobin but reduction in C-reactive protein. *Am J Clin Nutr*. 2008; 87(1): 114-125.

Yancy WS Jr, Westman EC, McDuffie JR, Grambow SC, Jeffreys AS, Bolton J, Chalecki A, Oddone EZ. A randomized trial of a low-carbohydrate diet vs orlistat plus a low-fat diet for weight loss. *Arch Intern Med.* 2010; 170(2): 136-145.

**Articles Suggested by Work Group Members**

Barnard ND, Gloede L, Cohen J, Jenkins DJA, Turner-McGrievy G, Green AA, Ferdowsian H. A low-fat vegan diet elicits greater macronutrient changes, but is comparable in adherence and acceptability, compared with a more conventional diabetes diet among individuals with type 2 diabetes. *J Am Diet Assoc*. 2009; 109: 263-272.

Brehm BJ, Lattin BL, Summer SS, Boback JA, Gilchrist GM, Jandacek RJ, D’Alessio DA. One-year comparison of a high-monounsaturated fat diet with a high-carbohydrate diet in type 2 diabetes. *Diabetes Care*. 2009; 32: 215-220.

Davis NJ, Tomuta N, Schechter C, Isasi CR, Segal-Isaacson CJ, Stein D, Zonszein J, Wylie-Rosett J. Comparative study of the effects of a one-year dietary intervention of a low-carbohydrate diet vs. a low-fat diet on weight and glycemic control in type 2 diabetes. *Diabetes Care*. 2009; 32: 1, 147-1, 152.

De Natale C, Annuzzi G, Bozzetto L, Mazzarella R, Costabile G, Ciano O, Riccardi G, Rivellese AA. Effects of a plant-based high-carbohydrate/high-fiber diet vs. high-monounsaturated fat/low-carbohydrate diet on postprandial lipids in type 2 diabetic patients. *Diabetes Care.* 2009; 32: 2, 168–2, 173.

Elhayany A, Lustman A, Abel R, Attal-Singer J, Vinker S. A low carbohydrate Mediterranean diet improves cardiovascular risk factors and diabetes control among overweight patients with type 2 diabetes mellitus: A one-year prospective randomized intervention study. *Diabetes Obes Metab.* 2010; 12: 204–209.

Esposito K, Maiorino MI, Ciotola M, Di Palo C, Scognamiglio P, Gicchino M, Petrizzo M, Saccomanno F, Beneduce F, Ceriello A, Giugliano D. Effects of a Mediterranean-style diet on the need for antihyperglycemic drug therapy in patients with newly diagnosed type 2 diabetes: A randomized trial. *Ann Intern Med.* 2009; 151: 306–314.

Haimoto H, Sasakabe T, Wakai K, Umegaki H. Effects of a low-carbohydrate diet on glycemic control in outpatients with severe type 2 diabetes. *Nutr Metab (Lond).* 2009; 6: 21.

Kirk JK, Graves DE, Craven TE, Lipkin EW, Austin M, Margolis KL. Restricted-carbohydrate diet in patients with type 2 diabetes: A meta-analysis. *J Am Diet Assoc*. 2008; 108: 91-100.

Kodama S, Saito K, Tanaka S, Maki M, Yachi Y, Sato M, Sugawara A, Totsuka K, Shimano H, Ohashi Y, Yamada N, Sone H. Influence of fat and carbohydrate proportions on the metabolic profile in patients with type 2 diabetes: A meta-analysis.*Diabetes Care*. 2009; 32: 959-965.

Nielsen JV, Joensson EA. Low-carbohydrate diet in type 2 diabetes: stable improvement of bodyweight and glycemic control during 44 months follow-up. *Nutr Metab (Lond)*. 2008; 5: 14-19.

Powers MA, Cuddihy RM, Wesley D, Morgan B. Continuous glucose monitoring reveals different glycemic responses of moderate- vs high-carbohydrate lunch meals in people with type 2 diabetes. *J Am Diet Assoc.* 2010; 110: 1, 912–1, 915.

Strychar IS, Cohn JS, Renier G, Rivard M, Aris-Jilwan N, Beauregard H, Meltzer S, Belanger A, Dumas R, Ishac A, Radwan F, Yale J-F. Effects of a diet higher in carbohydrate/lower in fat vs. lower in carbohydrate/higher in monounsaturated fat on postmeal triglyceride concentrations and other cardiovascular risk factors in type 1 diabetes. *Diabetes Care.* 2009; 32: 1, 597–1, 599.

Turner-McGrievy G, Barnard ND, Cohen J, Jenkins DJA, Gloede L, Green AA. Changes in nutrient intake and dietary quality among participants with type 2 diabetes following a low-fat vegan diet or a conventional diabetes diet for 22 weeks. *J Am Diet Assoc*. 2008; 108: 1, 636-1, 645.

Wheeler ML, Dunbar SA, Jaacks LM, Karmally W, Mayer-Davis EJ, Wylie-Rosett J, Yancy WS. Macronutrients, food groups and eating patterns in the management of diabetes: A systematic review of the literature, 2010. *Diabetes Care*. 2012; 35: 434–445.

**Inclusion List:**

List of Included Articles

Delahanty LM, Nathan DM, Lachin JM, Hu FB, Cleary PA, Ziegler GK, Wylie-Rosett J, Wexler DJ; Diabetes Control and Complications Trial/Epidemiology of Diabetes. Association of diet with glycated hemoglobin during intensive treatment of type 1 diabetes in the Diabetes Control and Complications Trial. *Am J Clin Nutr*. 2009; 89(2): 518-524.

Strychar IS, Cohn JS, Renier G, Rivard M, Aris-Jilwan N, Beauregard H, Meltzer S, Belanger A, Dumas R, Ishac A, Radwan F, Yale J-F. Effects of a diet higher in carbohydrate/lower in fat vs. lower in carbohydrate/higher in monounsaturated fat on postmeal triglyceride concentrations and other cardiovascular risk factors in type 1 diabetes. *Diabetes Care*. 2009; 32: 1, 597–1, 599.

Wolever TM, Gibbs AL, Mehling C, Chiasson JL, Connelly PW, Josse RG, Leiter LA, Maheux P, Rabasa-Lhoret R, Rodger NW, Ryan EA. The Canadian Trial of Carbohydrates in Diabetes (CCD), a one-year controlled trial of low-glycemic-index dietary carbohydrate in type 2 diabetes: No effect on glycated hemoglobin but reduction in C-reactive protein. *Am J Clin Nutr*. 2008; 87(1): 114-125.

**Included Articles 2006**

No articles from 2006 were included.

**Included Articles 2001**

No articles from 2001 were included.

**Articles Considered, but Excluded**

List of Excluded Articles with Reason

| **Article (A–L)** | **Reason for Exclusion** |
| --- | --- |
| Barnard ND, Gloede L, Cohen J, Jenkins DJA, Turner-McGrievy G, Green AA, Ferdowsian H. A low-fat vegan diet elicits greater macronutrient changes, but is comparable in adherence and acceptability, compared with a more conventional diabetes diet among individuals with type 2 diabetes. *J Am Diet Assoc*. 2009; 109: 263-272. | A 6% weight loss in vegan group and a4% weight loss in ADAgroup. |
| Brehm BJ, Lattin BL, Summer SS, Boback JA, Gilchrist GM, Jandacek RJ, D’Alessio DA. One-year comparison of a high-monounsaturated fat diet with a high-carbohydratediet in type 2 diabetes. *Diabetes Care*. 2009; 32: 215-220. | A 23% dropout rate. |
| Davis NJ, Tomuta N, Schechter C, Isasi CR, Segal-Isaacson CJ, Stein D, Zonszein J, Wylie-Rosett J. Comparative study of the effects of a one-year dietary intervention of a low-carbohydrate diet vs. a low-fat diet on weight and glycemic control in type 2 diabetes. *Diabetes Care*. 2009; 32: 1, 147-1, 152. | Moved to Weight Loss. |
| De Natale C, Annuzzi G, Bozzetto L, Mazzarella R, Costabile G, Ciano O, Riccardi G, Rivellese AA. Effects of a plant-based high-carbohydrate/high-fiber diet vs. high-monounsaturated fat/low-carbohydrate diet on postprandial lipids in type 2 diabetic patients. *Diabetes Care*. 2009; 32: 2, 168–2, 173. | Study duration was only four weeks long. |
| Elhayany A, Lustman A, Abel R, Attal-Singer J, Vinker S. A low-carbohydrate Mediterranean diet improves cardiovascular risk factors and diabetes control among overweight patients with type 2 diabetes mellitus: A one-year prospective randomized intervention study. *Diabetes Obes Metab.* 2010; 12: 204–209. | A 25% dropout rate. |
| Esposito K, Maiorino MI, Ciotola M, Di Palo C, Scognamiglio P, Gicchino M, Petrizzo M, Saccomanno F, Beneduce F, Ceriello A, Giugliano D. Effects of a Mediterranean-style diet on the need for antihyperglycemic drug therapy in patients with newly diagnosed type 2 diabetes: A randomized trial. *Ann Intern Med*. 2009; 151: 306–314. | A 4% to 7% weight loss in groups; participants assigned to the Mediterranean-style diet lost more weight. |
| Haimoto H, Iwata M, Wakai K, Umegaki H. Long-term effects of a diet loosely restricting carbohydrates on HbA1c levels, BMI and tapering of sulfonylureas in type 2 diabetes: A two-year follow-up study. *Diabetes Res Clin Pract.* 2008; 79(2): 350-356. | A 41% dropout rate; there was significant weight loss between groups. |
| Haimoto H, Sasakabe T, Wakai K, Umegaki H. Effects of a low-carbohydrate diet on glycemic control in outpatients with severe type 2 diabetes. *Nutr Metab (Lond).* 2009; 6: 21. | Non-controlled trial, class D. |
| Iqbal N, Vetter ML, Moore RH, Chittams JL, Dalton-Bakes CV, Dowd M, Williams-Smith C, Cardillo S, Wadden TA. Effects of a low-intensity intervention that prescribed a low-carbohydrate vs. a low-fat diet in obese. *Obesity (Silver Spring)*. 2010; 18: 1733-1, 738. | A 47% dropout rate. |
| Kamada C, Yoshimura H, Okumura R, Takahashi K, Iimuro S, Ohashi Y, Araki A, Umegaki H, Sakurai T, Yoshimura Y, Ito H; Japanese Elderly Diabetes Intervention Trial Study Group. Optimal energy distribution of carbohydrate intake for Japanese elderly patients with type 2 diabetes: The Japanese Elderly Intervention Trial. *Geriatr Gerontol Int.* 2012; 12(Suppl 1); 41-49. | Cross-sectional analysis of baseline data, class D. |
| Kirk JK, Graves DE, Craven TE, Lipkin EW, Austin M, Margolis KL. Restricted-carbohydrate diet in patients with type 2 diabetes: A meta-analysis. *J Am Diet Assoc*. 2008; 108: 91-100. | Meta-analysis. |
| Klupa T, Solecka I, Nowak N, Szopa M, Kiec-Wilk B, Skupien J, Trybul I, Matejko B, Mlynarski W, Malecki MT. The influence of dietary carbohydrate content on glycaemia in patients with glucokinase maturity-onset diabetes of the young. *J Int Med Res*. 2011; 39(6): 2, 296-2, 301. | Only seven subjects had maturity-onset diabetes of the young. |
| Kodama S, Saito K, Tanaka S, Maki M, Yachi Y, Sato M, Sugawara A, Totsuka K, Shimano H, Ohashi Y, Yamada N, Sone H. Influence of fat and carbohydrate proportions on the metabolic profile in patients with type 2 diabetes: A meta-analysis. *Diabetes Care*. 2009; 32: 959-965. | Meta-analysis. |
| Kohnert KD, Augstein P, Zander E, Heinke P, Peterson K, Freyse EJ, Hovorka R, Salzsieder E. Glycemic variability correlates strongly with postprandial beta-cell dysfunction in a segment of type 2 diabetic patients using oral hypoglycemic agents. *Diabetes Care.* 2009; 32(6): 1, 058-1, 062. | Cross-sectional study, class D. |
| Kollannoor-Samuel G, Chhabra J, Fernandez ML, Vega-Lopez S, Perez SS, Damio G, Calle MC, D'Agostino D, Perez-Escamilla R. Determinants of fasting plasma glucose and glycosylated hemoglobin among low income Latinos with poorly controlled type 2 diabetes. *J Immigr Minor Health.* 2011; 13(5): 809-817. | Cross-sectional study, class D. |
| Krebs JD, Elley CR, Parry-Strong A, Lunt H, Drury PL, Bell DA, Robinson E, Moyes SA, Mann JI. The Diabetes Excess Weight Loss (DEWL) Trial: A randomised controlled trial of high-protein vs. high-carbohydrate diets over two years in type 2 diabetes. *Diabetologia*. 2012; 55(4): 905-914. | A 30% dropout rate. |

| **Article (M–Z)** | **Reason for Exclusion** |
| --- | --- |
| Mannucci E, Pala L, Monami M, Da Vico L, Bardini G, Dicembrini I, Ciani S, Lamanna C, Marchionni N, Rotella CM. Glucagon-like peptide-1 response to meals and post-prandial hyperglycemia in Type 2 diabetic patients. *J Endocrinol Invest*. 2010; 33(3): 147-150. | Meal study. |
| Mohan V, Radhika G, Sathya RM, Tamil SR, Ganesan A, Sudha V. Dietary carbohydrates, glycaemic load, food groups and newly detected type 2 diabetes among urban Asian Indian population in Chennai, India (Chennai Urban Rural Epidemiology Study 59). *Br J Nutr.* 2009; 102(10): 1, 498-1, 506. | Cross-sectional study, class D. |
| Nielsen JV, Joensson EA. Low-carbohydrate diet in type 2 diabetes: Stable improvement of bodyweight and glycemic control during 44 months follow-up. *Nutr Metab (Lond)*. 2008; 5: 14-19. | A 7% weight loss, moved to Weight Loss. |
| Park SH, Lee KS, Park HY. Dietary carbohydrate intake is associated with cardiovascular disease risk in Korean: Analysis of the third Korea National Health and Nutrition Examination Survey (KNHANES III). *Int J Cardiol*. 2010; 139(3): 234-240. | Cross-sectional study, class D. |
| Powers MA, Cuddihy RM, Wesley D, Morgan B. Continuous glucose monitoring reveals different glycemic responses of moderate- vs high-carbohydrate lunch meals in people with type 2 diabetes. *J Am Diet Assoc*. 2010; 110: 1, 912–1, 915. | Meal study. |
| Samaha FF, Iqbal N, Seshadri P, Chicano KL, Daily DA, McGrory J, Williams T, Williams M, Gracely EJ, Stern L. A low-carbohydrate as compared with a low-fat diet in severe obesity. *N Engl J Med.* 2003; 348: 2, 074-2, 081. | Published in 2003; only 39% had type 2 diabetes. |
| Schmidt S, Finan DA, Duun-Henriksen AK, Jorgensen JB, Madsen H, Bengtsson H, Holst JJ, Madsbad S, Norgaard K. Effects of everyday life events on glucose, insulin and glucagon dynamics in continuous subcutaneous insulin infusion-treated type 1 diabetes: Collection of clinical data for glucose modeling. *Diabetes Technol Ther.* 2012; 14(3): 210-217. | Study duration was only 24 days long. |
| Stern L, Iqbal N, Seshadri P, Chicano KL, Daily DA, McGrory J, Williams T, Gracely EJ, Samaha FF. The effects of low-carbohydrate vs. conventional weight loss diets in severely obese adults: One-year follow-up of a randomized trial. *Ann Intern Med*. 2004; 140: 778-785. | Published in 2004; only 83% had type 2 diabetes. |
| Taru C, Tsutou A, Nakawatase Y, Usami M, Miyawaki I. Gender differences of dietary self-management behavior affecting control indices in type II diabetes. *Kobe J Med Sci.* 2008; 54(2): E82-E96. | Cross-sectional study, class D. |
| Turner-McGrievy G, Barnard ND, Cohen J, Jenkins DJA, Gloede L, Green AA. Changes in nutrient intake and dietary quality among participants with type 2 diabetes following a low-fat vegan diet or a conventional diabetes diet for 22 weeks. *J Am Diet Assoc*. 2008; 108: 1, 636-1, 645. | A 6% weight loss in vegan group, a 4% weight loss in ADA group. |
| Vaccaro JA, Huffman FG. Monounsaturated fatty acid, carbohydrate intake, and diabetes status are associated with arterial pulse pressure. *Nutr J.* 2011; 10: 126. | Cross-sectional study, class D. |
| Westman EC, Yancy WS Jr, Mavropoulos JC, Marquart M, McDuffie JR. The effect of a low-carbohydrate, ketogenic diet vs. a low-glycemic index diet on glycemic control in type 2 diabetes mellitus. *Nutr Metab (Lond).* 2008; 5: 36. | A 42% dropout rate. |
| Wheeler ML, Dunbar SA, Jaacks LM, Karmally W, Mayer-Davis EJ, Wylie-Rosett J, Yancy WS. Macronutrients, food groups and eating patterns in the management of diabetes: A systematic review of the literature, 2010. *Diabetes Care.* 35: 434–445, 2, 012. | Systematic review. |
| Xu J, Eilat-Adar S, Loria CM, Howard BV, Fabsitz RR, Begum M, Zephier EM, Lee ET. Macronutrient intake and glycemic control in a population-based sample of American Indians with diabetes: The Strong Heart Study. *Am J Clin Nutr*. 2007; 86(2): 480-487. | Cross-sectional study, class D. |
| Yancy WS Jr, Westman EC, McDuffie JR, Grambow SC, Jeffreys AS, Bolton J, Chalecki A, Oddone EZ. A randomized trial of a low-carbohydrate diet vs. orlistat plus a low-fat diet for weight loss. *Arch Intern Med*. 2010; 170(2): 136-145. | Only 32% had type 2 diabetes. |

**Excluded Articles 2006** 

| **Article** | **Reason for Exclusion** |
| --- | --- |
| Allick G, Bisschop PH, Ackermans MT, Endert E, Meijer AJ, Kuipers F, Sauerwein HP, Romijn JA. A low-carbohydrate/high-fat diet improves glucoregulation in type 2 diabetes mellitus by reducing postabsorptive glycogenolysis. *J Clin Endocrinol Metab.* 2004; 89(12): 6, 193-6, 197. | N for treatment groups was less than 10 each. |
| Boden G, Sargrad K, Homko C, Mozzoli M, Stein TP. Effect of a low-carbohydrate diet on appetite, blood glucose levels, and insulin resistance in obese patients with type 2 diabetes. *Ann Intern Med*. 2005; 142(6): 403-411. | Study duration was only 14 days long. |
| Daly ME, Paisey R, Paisey R, Millward BA, Eccles C, Williams K, Hammersley S, MacLeod KM, Gale TJ. Short-term effects of severe dietary carbohydrate restriction advice in Type 2 diabetes: A randomized controlled trial. *Diabet Med.* 2006; 23(1): 15-20. | A 22% dropout rate. |
| Gerhard GT, Ahmann A, Meeuws K, McMurry MP, Duell PB, Connor WE. Effects of a low-fat diet compared with those of a high monounsaturated fat diet on body weight, plasma lipids and lipoproteins, and glycemic control in type 2 diabetes. *Am J Clin Nutr*. 2004; 80(3): 668-673. | Study duration was only six weeks long. |
| Komiyama N, Kaneko T, Sato A, Sato W, Asami K, Onaya T, Tawata M. The effect of high carbohydrate diet on glucose tolerance in patients with type 2 diabetes mellitus. *Diabetes Res Clin* *Pract*. 2002; 57(3): 163-170. | Study duration was only seven days. |
| Nielsen JV, Jonsson E, Ivarsson A. A low carbohydrate diet in type 1 diabetes: Clinical experience—a brief report. *Ups J Med Sci*. 2005; 110(3): 267-273. | Of 24 subjects, only 15 were included in the analysis. Actual weight loss was not reported, but the article mentions that two subjects lost weight due to other illnesses and all seven overweight patients lost weight. Normal-weight patients were weight stable. |
| Nielsen JV, Jonsson E, Nilsson AK. Lasting improvement of hyperglycemia and body weight: Low-carbohydrate diet in type 2 diabetes. A brief report. *Ups J Med Sci*. 2005; 110(2): 179-183. | A 11% weight loss in low-carbohydrate diet group. |
| Rosenfalck AM, Almdal T, Viggers L, Madsbad S, Hilsted J. A low-fat diet improves peripheral insulin sensitivity in patients with type 1 diabetes. *Diabet Med*. 2006; 23(4): 384-392. | A 24% dropout rate. |
| Sargrad KR, Homko C, Mozzoli M, Boden G. Effect of high protein vs. high carbohydrate intake on insulin sensitivity, body weight, hemoglobin A1c, and blood pressure in patients with type 2 diabetes mellitus. *J Am Diet Assoc*. 2005; 105(4): 573-580. | N for treatment groups was less than 10 each. |

**Excluded Articles 2001** 

| **Article** | **Reason for Exclusion** |
| --- | --- |
| American Diabetes Association Position Paper. Evidence-based nutrition principles and recommendations for the treatment and prevention of diabetes and related complications. *Diabetes Care.* 2002; 25(suppl2). | Position paper, not used. |
| Gannon MC, Nuttall FQ, Westphal SA, Fang D, Ercan-Fang N. Acute metabolic response to high-carbohydrate, low-starch meals in subjects with type 2 diabetes. *Diabetes Care.* 1998;1, 619-1, 626. | N for treatment groups was less than 10 each. |
| Garg A, Bantle JP, Henry RR, Coulston AM, Griver KA, Raatz SK, Brinkley L, Chen Y-DI, Grundy SM, Huet BA, Reaven GM. Effects of varying carbohydrate content of diet in patients with non-insulin dependent diabetes mellitus. *JAMA*. 1994; 271: 1, 421-1, 428. | Study duration was only six weeks long. |
| Parillo M, Giacco R, Ciardullo AV, Rivellese AA, Riccardi G. Does a high-carbohydrate diet have different effects in NIDDM patients treated with diet alone or hypoglycemic drugs. *Diabetes Care.* 1996; 19: 498-500. | N for treatment groups was less than 10 each. |

Summary of Articles Identified to Review

**Number of Included Primary Research Articles Identified from all sources:** 3

**Number of Included Review Articles Identified from all sources:** 0

**Total Number of Included Articles:** 3

**Number of Articles Considered but Excluded:** 31

**Total Number of Articles Considered:** 12
