# Supplementary material for: Using risk of bias domains to identify opportunities for improvement in food- and nutrition-related research: An evaluation of research type and design, year of publication, and source of funding
Source: PLoS One. 2018 Jul 5;13(7):e0197425. doi: 10.1371/journal.pone.0197425 (PMC6033375; doi:10.1371/journal.pone.0197425)
Supplement: S3 Fig — (PDF) [file pone.0197425.s005.pdf]

S-3 Figure. Example of search strategy and flow from AHRQ Reports

Page 15 and 16 and page 22 are excerpted from:

MacLean CH, Mojica, WA, Morton SC, Pencharz J, Hasenfeld Garland R, Tu W, Newberry SJ, Jungvig LK, Grossman J, Khanna P, Rhodes S, Shekelle P. Effects of Omega-3 Fatty Acids on Lipids and Glycemic Control in Type II Diabetes and the Metabolic Syndrome and on Inflammatory Bowel Disease, Rheumatoid Arthritis, Renal Disease, Systemic Lupus Erythematosus, and Osteoporosis. Evidence Report/Technology Assessment. No. 89 (Prepared by Southern California/RAND Evidence-based Practice Center, under Contract No. 290-02-0003). AHRQ Publication No. 04-E012-2. Rockville, MD: Agency for Healthcare Research and Quality. March 2004.

Identification of Literature Sources

Potential evidence for our study came from three sources: on-line library databases, the reference lists of all relevant articles, and industry experts.

Jessie McGowan, Senior Information Scientist, and Nancy Santesso, Knowledge Translation Specialist, at the University of Ottawa were responsible for developing a common search strategy for omega-3 fatty acids for the 3 participating EPCs. Nancy Santesso developed a core omega-3 search strategy in collaboration with project librarians, biochemists, nutritionists, and clinicians, who also provided biochemical names, abbreviations, food sources, and commercial product names for omega-3 fatty acids. The literature search was not restricted by language of publication or by study design, except with the MeSH term, “dietary fats,” in order to increase specificity. When possible, the searches were limited to studies involving human subjects. The core search strategy is detailed in Appendix A.3.

For the SCEPC, this core search strategy was incorporated into 6 specific searches that focused on our relevant disease categories: rheumatoid arthritis, bone density, SLE, renal disease, diabetes, and gastrointestinal diseases. The strategies for these searches are detailed in Appendix A.3.

The following databases were searched: Medline (1966-July, 2003), Premedline (July 8, 2003), Embase (1980-Week 27, 2003), Cochrane Central Register of Controlled Trials (2nd Quarter, 2003), CAB Health (1973-June 2003), Dissertation Abstracts (1861-to December 2002). All of these databases were searched using the Ovid interface, except CAB Health, which was searched through SilverPlatter. Any duplicate records were identified and removed within each search question using Reference Manager software, except for the last update, which was imported into EndNote. The citations obtained from these literature searches were sent to the SCEPC via e-mail.

The citations were transferred to a secured Internet-based software system (termed D2D) that enabled us to view article titles and abstracts electronically. Two reviewers, Walter Mojica and James Pencharz, used the computerized software system to independently evaluate the citations and abstracts using the review form in Figure B.1, Appendix B, which was loaded onto the computerized system.

The reviewers flagged article titles that focused on omega-3 fatty acids and any of the following disease conditions: diabetes mellitus, inflammatory bowel disease (ulcerative colitis and Crohn's disease), rheumatoid arthritis, SLE, renal disease, osteoporosis, or bone mineral status. In addition, they flagged article titles that pertained to the disease conditions of the other participating EPCs (i.e., cardiovascular disease or asthma). Language was not a barrier to inclusion. Articles that either reviewer flagged were ordered, as well as those articles in which it was unclear from the title or abstract whether the article was relevant. The articles were ordered from the RAND library, the UCLA library, or Kessler-Hancock, a San Francisco-based literature retrieval firm with contacts around the world. The literature was tracked using ProCite and Access software.

In addition, we sent letters to industry experts recommended by the Office Dietary Supplements to obtain any unpublished data (Figure A.3.1).

Figure 3.1. Literature flow.

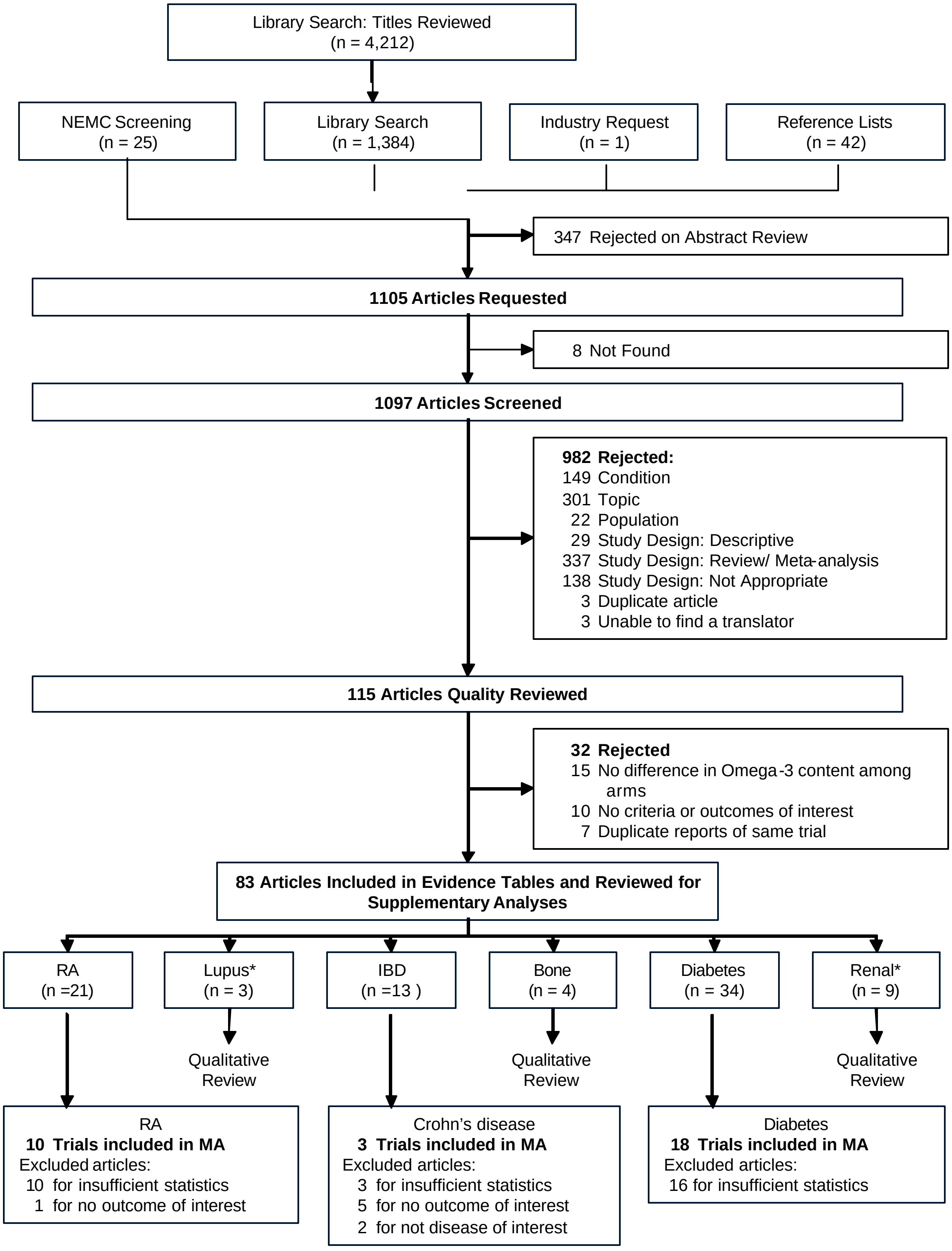

* One article reported both lupus and renal outcomes.
